# Few-layer bismuth selenides exfoliated by hemin inhibit amyloid-β_1–42_ fibril formation

**DOI:** 10.1038/srep10171

**Published:** 2015-05-28

**Authors:** Jian Peng, Yunjing Xiong, Zhiqin Lin, Liping Sun, Jian Weng

**Affiliations:** 1 College of Chemistry and Chemical Engineering, Xiamen University, Xiamen 361005, P.R. China; 2 College of Materials, Xiamen University, Xiamen 361005, P.R. China; 3 ShenZhen Research Institute of Xiamen University, Shenzhen 518057, China

## Abstract

Inhibiting amyloid-β (Aβ) fibril formation is the primary therapeutic strategy for Alzheimer’s disease. Several small molecules and nanomaterials have been used to inhibit Aβ fibril formation. However, insufficient inhibition efficiency or poor metabolization limits their further applications. Here, we used hemin to exfoliate few-layer Bi_2_Se_3_ in aqueous solution. Then we separated few-layer Bi_2_Se_3_ with different sizes and thicknesses by fractional centrifugation, and used them to attempt to inhibit Aβ_1-42_ aggregation. The results show that smaller and thinner few-layer Bi_2_Se_3_ had the highest inhibition efficiency. We further investigated the interaction between few-layer Bi_2_Se_3_ and Aβ_1-42_ monomers. The results indicate that the inhibition effect may be due to the high adsorption capacity of few-layer Bi_2_Se_3_ for Aβ_1−42_ monomers. Few-layer Bi_2_Se_3_ also decreased Aβ-mediated peroxidase-like activity and cytotoxicity according to *in vitro* neurotoxicity studies under physiological conditions. Therefore, our work shows the potential for applications of few-layer Bi_2_Se_3_ in the biomedical field.

Alzheimer’s disease (AD), one of the leading causes of death in the world^1^, is a devastating neurodegenerative disorder and the most common form of dementia among people over the age of sixty-five. Senile plaques formed by the aggregation of Aβ peptides and neurofibrillary tangles comprised of primarily hyperphosphorylated tau in the brain are considered the pathologic hallmarks in AD^2^. According to the “Aβ hypothesis”, Aβ is generated from specific β- and γ-secretase cleavages of amyloid precursor protein^3^,^4^. Aβ-protein (1–40) (Aβ_1-40_) and Aβ-protein (1–42) (Aβ_1−42_), which only differ in the two extra C-terminal residues, are the main component of extracellular senile plaques. Though Aβ_1−40_ is 10 times more abundant than Aβ_1−42_, Aβ_1−42_ aggregates faster and plays an important role in AD development^5^,^6^. Aβ_1−42_ with a molecular weight of 4.5 kDa is an amphipathic polypeptide, which is prone to aggregate and form fibrils^7^. It is reported that the aggregation and deposition of Aβ_1−42_ in the brain is implicated in the aetiology of AD^8^, and inhibiting Aβ aggregation has been thought as one of the primary therapeutic strategies for AD^9^,^10^. Therefore, screening Aβ inhibitors is very important in the research of AD. However, it remains a challenge to find an effective inhibitor for Aβ aggregation.

There are several publications that suggest some small molecules might serve as Aβ inhibitors to prevent amyloid fibril formation. Potential inhibitors with different structures have been reported, such as peptide fragments^11^, organic dyes^12^ and small aromatic compounds^13^,^14^,^15^. However, there are several challenges for small molecules to inhibit Aβ aggregation^16^. First, the interactions between protein and small molecule may be weak^17^, resulting in insufficient efficiency to inhibit Aβ aggregation^18^. Second, small molecules may be accommodated by the highly plastic nature of protein surfaces, thus decreasing inhibition efficiency^19^. To solve these problems, new Aβ inhibitors are in urgent demand. Interestingly, nanomaterials have been used to inhibit Aβ fibril formation. Among them gold nanoparticles (NPs)^9^,^20^, magnetic NPs^21^, quantum dots^22^, polymeric NPs^23^, graphene oxide^24^, carbon nanotubes^25^ and polyoxometalate with a Wells–Dawson structure^26^ have been used to inhibit Aβ fibril formation. However, there are also several challenges for nanomaterials to serve as Aβ inhibitors. The first problem is that these nanomaterials cannot be degraded, and are poorly metabolized. The second problem is that most of them fail to reduce Aβ-mediated neurotoxicity and peroxidase-like activity^27^,^28^. Furthermore, their inhibition mechanism has not been fully understood. To solve these problems, it is important to find new biocompatible materials that could be used to effectively inhibit Aβ aggregation.

Bismuth selenide (Bi_2_Se_3_), a topological insulator, has attracted wide interest in condensed matter physics due to the unique surface electronic states^29^,^30^,^31^. It consists of stacked layers of a laminated structure held together by weak van der Waals interactions. The three-dimensional (3D) structure restricts its application due to the bulk state of high carrier density^32^. Thus, the production of two-dimensional (2D) Bi_2_Se_3_ from its 3D bulk materials is in urgent demand in order to acquire superior property for potential applications. Up to now, 2D nanomaterials were mainly prepared by bottom-up synthesis and top-down exfoliation^33^. 3D materials with weak van der Waals forces can be exfoliated into thin flakes by mechanical or chemical exfoliation^34^,^35^, which is a top-down process. This method has been used to produce single-layer or few-layer 2D materials such as graphene and few-layer molybdenum sulfide because it is easier and more convenient than other methods^36^,^37^. Therefore, we try to prepare few-layer Bi_2_Se_3_ by exfoliation of bulk Bi_2_Se_3_ in solution. Though 2D few-layer Bi_2_Se_3_ shows excellent properties which can be compared with graphene, there are only a few publications on the biomedical applications of Bi_2_Se_3_^38^,^39^,^40^,^41^. At the same time, the Se element can inhibit reactive oxygen species^42^. The Bi element, with atomic number 83, has a high photoelectric absorption coefficient and may be used as a cancer radio-sensitizer and X-ray contrast agent. Therefore, Bi_2_Se_3_ has been reported to serve as a theranostic reagent for simultaneous cancer imaging and therapy^40^. More importantly, it has been reported that Bi_2_Se_3_ nanoplates show low toxicity for mice even at high doses of 20 mg kg^-1^. More surprisingly, Bi_2_Se_3_ nanoplates can be metabolized after long-term toxicological responses^41^. These properties of 2D Bi_2_Se_3_ stimulated us to investigate the interaction of 2D Bi_2_Se_3_ and Aβ, and explore its ability to inhibit Aβ fibril formation.

In order to combine the two advantages of 2D nanomaterials and small molecules in inhibiting Aβ fibril formation, we prepared few-layer Bi_2_Se_3_ coated by small molecules and investigated its ability to inhibit Aβ fibril formation. Here, few-layer Bi_2_Se_3_ exfoliated by hemin (Supplementary Fig. S1) was used to inhibit Aβ fibril formation under near-physiological conditions *in vitro* with satisfactory results. The preparation and application of exfoliated few-layer Bi_2_Se_3_ are simple, low cost and environmental-friendly. These advantages indicate that our present work could provide new insights into the potential application of 2D few-layer Bi_2_Se_3_ in medicine and biotechnology.

## Results

### Preparation and characterization of bulk Bi_2_Se_3_

**Figure 1:**
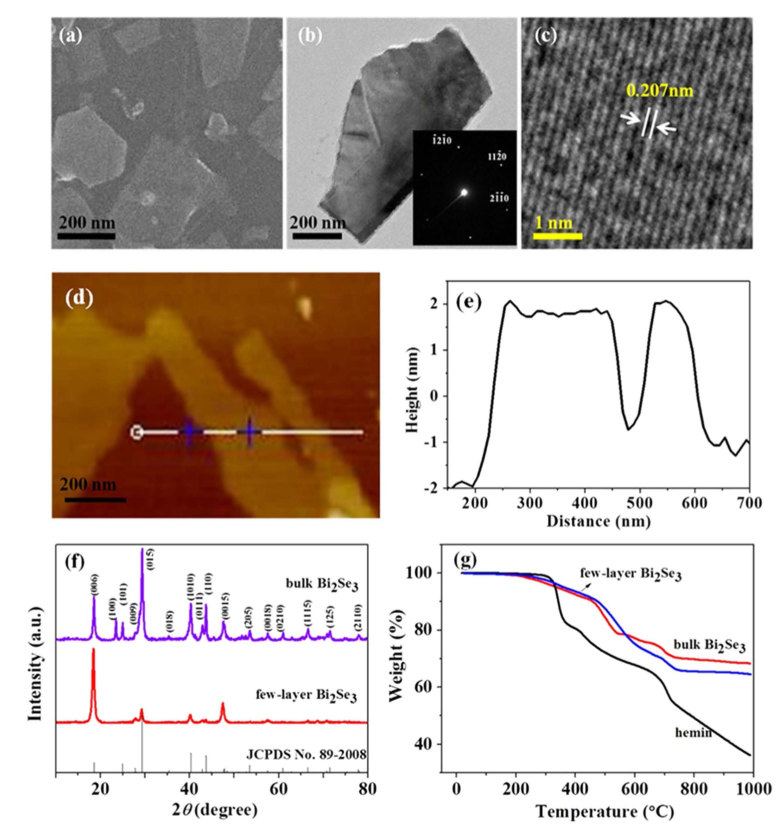
Few-layer Bi_2_Se_3_exfoliated with hemin (0.05 mg mL^-1)^ in 0.1% NH_3_·H_2_O. (a) SEM image of few-layer Bi_2_Se_3_. (b) TEM image of few-layer Bi_2_Se_3_, insert: SAED pattern of few-layer Bi_2_Se_3_. (c) HRTEM image of few-layer Bi_2_Se_3_. (d, e) AFM image and the corresponding height profile of few-layer Bi_2_Se_3_. (f) XRD patterns of bulk and few-layer Bi_2_Se_3_. (g) TGA curves for hemin, bulk Bi_2_Se_3_ and few-layer Bi_2_Se_3_ in N_2_ atmosphere with a ramp of 10°C min^-1^.

Bulk Bi_2_Se_3_ was prepared by hydrothermal synthesis. The as-synthesized bulk Bi_2_Se_3_ exhibits sheet-like structure with a wide size distribution and is inclined to aggregate together, which was confirmed by scanning electron microscopy (SEM) and transmission electron microscopy (TEM) (Supplementary Fig. S2a, b). The selected area electron diffraction (SAED) pattern (Supplementary Fig. S2c) showed that Bi_2_Se_3_ was indexed as a 6-fold symmetry [001] zone axis pattern, which is consistent with the layered structure along the z axis. Then, energy dispersive X-ray (EDX) spectrum was employed to confirm the elementary composition of Bi_2_Se_3_ (Supplementary Fig. S2d). Furthermore, the thickness of bulk Bi_2_Se_3_ was about 50 nm, as-determined by atomic force microscopy (AFM, Supplementary Fig. S2e). Finally, the as-synthesized bulk Bi_2_Se_3_ was investigated by X-ray diffraction (XRD, Fig. 1f). All the labeled peaks were readily indexed as rhombohedral Bi_2_Se_3_ (JCPDS no. 89-2008).

### Optimization of exfoliation conditions

The as-prepared bulk Bi_2_Se_3_ was firstly dispersed in hemin dissolved in 0.1% ammonia water (NH_3_·H_2_O) solution. Then the mixture was sonicated for about 40 h to form few-layer Bi_2_Se_3_. Hemin, an iron-containing porphyrin small molecule, plays an important role in dispersing few-layer Bi_2_Se_3_. Therefore, it was necessary to investigate the effect of hemin concentration on the yield of few-layer Bi_2_Se_3_ (Supplementary Fig. S3). The results showed that the optimal concentration of hemin was 0.05 mg mL^-1^. NH_3_·H_2_O solution also plays an important role in dissolving hemin, which can affect the yield of few-layer Bi_2_Se_3_ (Supplementary Fig. S4a, b). 0.1% is the optimal concentration of NH_3_·H_2_O. We further explored the effect of pH on the yield of few-layer Bi_2_Se_3_ (Supplementary Fig. S4c, d). The pH was adjusted by 0.1 M sodium hydroxide (NaOH) solution to substitute NH_3_·H_2_O and 10 was the optimal pH that was close to the pH of 0.1% NH_3_·H_2_O. We owe the exfoliation of bulk Bi_2_Se_3_ materials to the energy provided by the ultrasound waves, which overcome the van der Waals forces between Bi_2_Se_3_ layers. Therefore, the ultrasonic time is also an important parameter that should be investigated (Supplementary Fig. S5). The yield of few-layer Bi_2_Se_3_ was improved with increasing ultrasonic time; however, above an ultrasonic time of 40 h there was little change in the yield of few-layer Bi_2_Se_3_, and as such 40 h was chosen as the optimal ultrasonic time.

### Characterization of few-layer Bi_2_Se_3_

Ultraviolet-visible (UV-vis) absorption spectra of the mixture of hemin and Bi_2_Se_3_ before and after sonication were measured, respectively (Supplementary Fig. S6a). Before sonication, the solution exhibited a brown color and the spectrum of the mixture had a strong peak at 388 nm attributed to the Soret band of hemin, as well as a group of weak peaks between 500 and 700 nm ascribed to the Q-bands of hemin^43^. After sonication of 40 h, the color of solution changed from brown to gray (Supplementary Fig. S6b), maximum absorption band of the Soret band of hemin was red-shifted from 388 to 403 nm and a broad absorption in the visible light region appeared. These results are similar to few-layer Bi_2_Se_3_ exfoliated by N-methyl-2-pyrrolidone in our previous work^44^, which could be attributed to the formation of few-layer Bi_2_Se_3_. At the same time, the absorption also increased gradually as the sonication time was extended (Supplementary Fig. S5), which revealed that more few-layer Bi_2_Se_3_ could be obtained with increasing sonication time. This suggests that the absorption was as a result of few-layer Bi_2_Se_3_.

The as-obtained few-layer Bi_2_Se_3_ was a thin 2D flake according to the SEM (Fig. 1a) and TEM images (Fig. 1b). Furthermore, according to the SAED pattern (Fig. 1b insert), few-layer Bi_2_Se_3_ was indexed as a 6-fold symmetry [001] zone axis pattern, which is consistent with the layered structure along the z axis. Also, it revealed the single-crystalline nature of the thin 2D flake. The distance between the adjacently hexagonal lattice fringes investigated by the HRTEM was 0.207 nm for Bi_2_Se_3_ (Fig. 1c), which is consistent with the lattice space of the (110) plane. The AFM image (Fig. 1d) also showed the flake structure and the thickness of exfoliated Bi_2_Se_3_ was 3-4 nm (Fig. 1e), which nearly equals to 3-4 layers of Bi_2_Se_3_^45^. The XRD pattern (Fig. 1f) of few-layer Bi_2_Se_3_ showed a high degree of [001] orientation and some characteristic peaks disappeared compared to bulk Bi_2_Se_3_, which indicated that bulk Bi_2_Se_3_ had been successfully exfoliated. To further confirm the exfoliation of Bi_2_Se_3_, Raman spectrum was employed (Supplementary Fig. S7). The A^1^_1*g*_ mode of few-layer Bi_2_Se_3_ produced a red shift compared with that of bulk Bi_2_Se_3_, which could be attributed to the phonon softening^46^. Furthermore, the content of hemin in few-layer Bi_2_Se_3_ was 11.8%, as calculated by thermogravimetric (TGA) analysis (Fig. 1g), which is consistent with the calculated value (12.0%) by X-ray photoelectron spectroscopy (XPS) and EDX (Supplementary Fig. S8).

### Preparation of few-layer Bi_2_Se_3_ with different thicknesses

Fractional centrifugation was employed here to obtain few-layer Bi_2_Se_3_ with different layers. Firstly, the few-layer Bi_2_Se_3_ was characterized by UV-vis absorption spectra (Supplementary Fig. S9). Interestingly, we found that the dispersion solutions of few-layer Bi_2_Se_3_ produced a broad absorption in the visible light region compared to bulk Bi_2_Se_3_. Furthermore, few-layer Bi_2_Se_3_ stock solutions handled at different centrifugal speeds demonstrated different UV-vis absorption spectra. With centrifugal speed increasing from 2000 to 13000 rpm, the maximum absorption band of sample was blue-shifted gradually from 570 to 400 nm, resulting from quantum size effect^44^, which indicated the production of few-layer Bi_2_Se_3_ with different sizes and layers.

**Figure 2:**
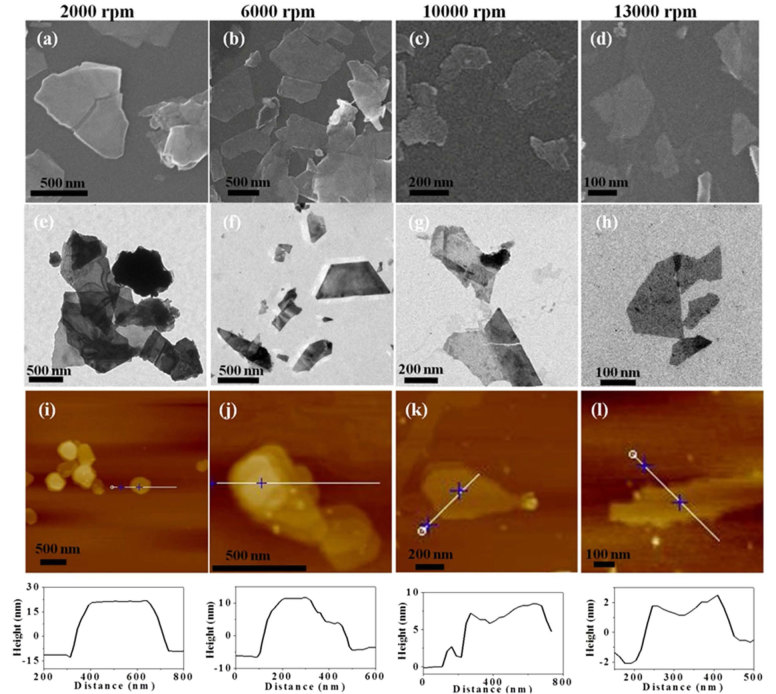
Few-layer Bi_2_Se_3_ with different layers. SEM, TEM and AFM images of few-layer Bi_2_Se_3_ stock solutions handled at 2000 (a, e, i), 6000 (b, f, j), 10000 (c, g, k) and 13000 rpm (d, h, l), respectively.

The size distribution and corresponding height profile of few-layer Bi_2_Se_3_ collected at different centrifugation speeds were distinctive. TEM and SEM were used to measure the sizes of few-layer Bi_2_Se_3_ (Fig. 2). With centrifugal speed increasing from 2000 to 13000 rpm, the size of sample decreased gradually from 637 ± 183 to 105 ± 31 nm, indicating the production of few-layer Bi_2_Se_3_ with different sizes (Table 1). AFM is frequently used to character 2D materials. Here we used it to investigate the thickness of few-layer Bi_2_Se_3_. With centrifugal speed increasing from 2000 to 13000 rpm, the thickness of few-layer Bi_2_Se_3_ decreased from about 40 to 3 nm (Fig 2.i-l), which further indicated the production of few-layer Bi_2_Se_3_ with different layers.

### Inhibiting Aβ_1-42_ fibril formation by few-layer Bi_2_Se_3_

**Figure 3:**
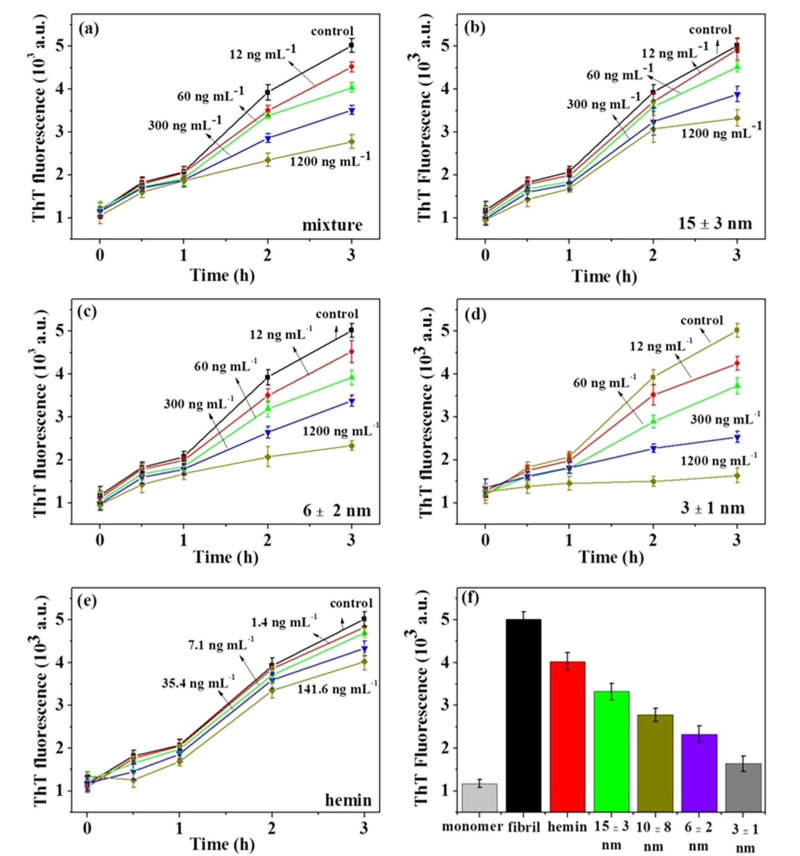
Inhibiting Aβ_1-42_ fibril formation by few-layer Bi_2_Se_3_ with different thicknesses investigating by ThT assay. Kinetics of Aβ_1-42_ fibril formation at 37°C in presence of few-layer Bi_2_Se_3_ with thickness of 10 ± 8 nm (mixture, a) , 15 ± 3 nm (b), 6 ± 2 nm (c), 3 ± 1 nm (d) and hemin (e). (f) ThT fluorescence intensity of Aβ_1-42_ monomer, Aβ_1-42_ fibril, Aβ_1-42_ incubated at 37°C for 3 h in presence of hemin and few-layer Bi_2_Se_3_ with different thicknesses, respectively. Fluorescence emissions were monitored at 485 nm with excitation at 442 nm. Three replicates were performed.

Thioflavine T (ThT) is a classic amyloid dye that is frequently used to probe Aβ fibril formation due to its strong fluorescence emission upon binding to cross-β fibril structures^47^,^48^,^49^. We co-incubated Aβ_1-42_ monomer and few-layer Bi_2_Se_3_ with different concentrations, and then monitored Aβ fibril formation kinetics by ThT fluorescence assay. Modified Krebs-Henseliet buffer, which mimics near-physiological conditions^8^, was used in the following experiments except where specifically noted. Aβ_1-42_ fibril formation in modified Krebs-Henseliet buffer without few-layer Bi_2_Se_3_ was firstly investigated by ThT fluorescence assay (Supplementary Fig. S10). In the absence of few-layer Bi_2_Se_3_, Aβ_1-42_ formed ThT-positive β-sheets instantaneously and ThT fluorescence reached maximum intensity at 3 h and then decreased gradually. Therefore, 3 h was selected as the appropriate time to study the effect of few-layer Bi_2_Se_3_ on Aβ_1-42_ fibril formation. The Aβ fibril formation kinetics in the absence and presence of few-layer Bi_2_Se_3_ with different thicknesses are shown in Fig. 3. Before fractional centrifugation, few-layer Bi_2_Se_3_ with a wide thickness distribution (10 ± 8 nm) was named a mixture. When the mixture was introduced (Fig. 3a), the fluorescence intensity at 3 h gradually decreased with increasing concentration of few-layer Bi_2_Se_3_, indicating consistent inhibition of Aβ_1-42_ fibril formation by few-layer Bi_2_Se_3_ in a dose-dependent manner. To further investigate the effect of few-layer Bi_2_Se_3_ thickness on Aβ_1-42_ fibril formation, few-layer Bi_2_Se_3_ with different thicknesses were introduced (Fig. 3b-d). Similarly, fluorescence intensities at 3 h gradually decreased with increasing concentration of few-layer Bi_2_Se_3_. It is interesting that the fluorescence intensity at 3 h gradually decreased with decreasing thickness of few-layer Bi_2_Se_3_ at a same concentration, indicating the inhibition efficiency increased with decreasing layers of few-layer Bi_2_Se_3_ (Table 1 and Fig. 3f). Few-layer Bi_2_Se_3_ contains 11.8% hemin calculated by TGA and XPS data (Supplementary Fig. S8). In order to investigate the effect of hemin on Aβ_1-42_ fibril formation, the corresponding hemin in few-layer Bi_2_Se_3_ with different concentrations was calculated and incubated with Aβ_1-42_ monomer in similar conditions (Fig. 3e and Table 1). Compared with few-layer Bi_2_Se_3_, the decrease of ThT fluorescence intensity induced by hemin was negligible. Therefore, the high inhibition efficiency of few-layer Bi_2_Se_3_ resulted mainly from few-layer Bi_2_Se_3_ (Fig. 3f, Table 1). Finally, the end-point ThT intensities at 3 h versus different Aβ inhibitors were plotted (Fig. 3f) and few-layer Bi_2_Se_3_ of 3 ± 1 nm had the best efficiency in inhibiting Aβ fibril formation. Therefore, few-layer Bi_2_Se_3_ of 3 ± 1 nm was used in following experiments except where specifically noted.

To confirm that the reduced ThT fluorescence intensity resulted from the inhibiting Aβ_1-42_ fibril formation by few-layer Bi_2_Se_3_, but not quenched by few-layer Bi_2_Se_3_ and hemin themselves, we investigated the effect of few-layer Bi_2_Se_3_ and hemin on ThT fluorescence intensity without Aβ_1-42_ monomer (Supplementary Fig. S11). The change of ThT intensity after incubation with few-layer Bi_2_Se_3_ and hemin is unobvious. At the same time, the intrinsic fluorescence of hemin can also be neglected compared to ThT fluorescence of Aβ_1-42_ fibril when the excitation wavelength is 442 nm (Supplementary Fig. S12). The results further suggest that the reduced ThT fluorescence intensity resulted from the inhibition of Aβ_1-42_ fibril formation by few-layer Bi_2_Se_3_.

**Figure 4:**
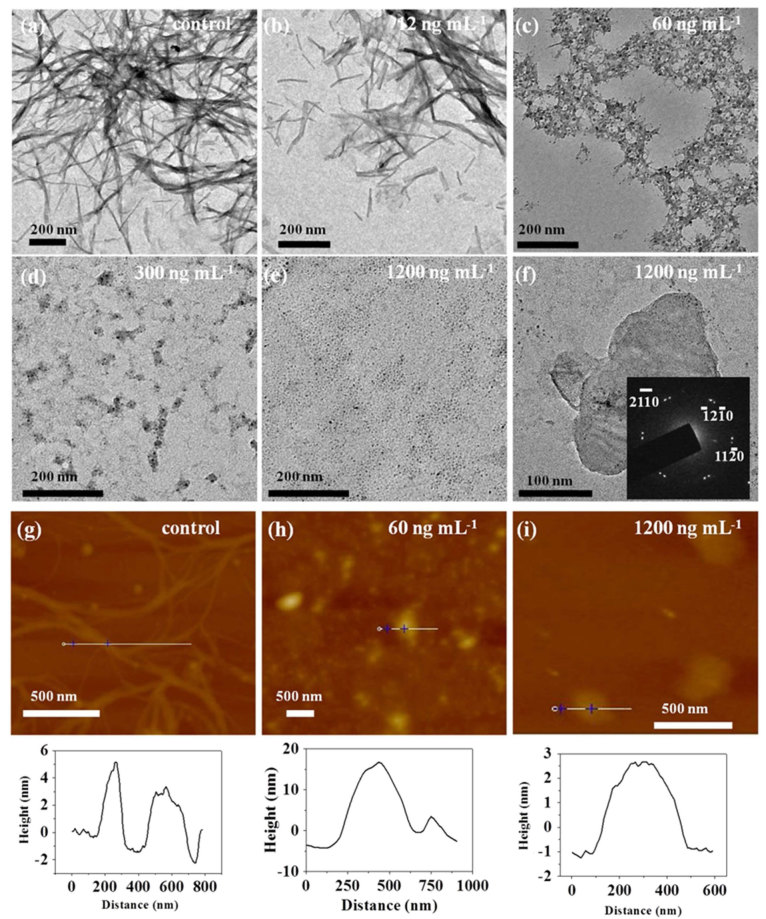
Few-layer Bi_2_Se_3_ inhibits Aβ_1-42_ fibril formation monitored by TEM and AFM. Morphologies of the Aβ_1-42_ species with and without few-layer Bi_2_Se_3_ (thickness, 3 ± 1 nm) with different concentrations monitored by TEM (a-f) and AFM (g-i). Insert in (f): SAED pattern of few-layer Bi_2_Se_3_. Corresponding height profiles of AFM were given below AFM. The concentration of few-layer Bi_2_Se_3_ and the scale bars were indicated in each image.

To further confirm the results of the ThT fluorescence assay, TEM and AFM were used to observe the morphologies of end-point products at 3 h (Fig. 4). After incubation of Aβ_1-42_ monomer without few-layer Bi_2_Se_3_ at 37°C for 3 h, Aβ_1-42_ formed long, smooth, and entangled fibrils as expected (Fig. 4a, g). However, after incubation of Aβ_1-42_ monomer and few-layer Bi_2_Se_3_ with different concentrations, different Aβ species were observed. In the presence of 12 ng mL^-1^ few-layer Bi_2_Se_3_ (Fig. 4b), fewer negatively-stained fibrils were observed and generally shorter in length than that without few-layer Bi_2_Se_3_. Aβ_1-42_ in the presence of 60 ng mL^-1^ few-layer Bi_2_Se_3_ formed some negatively-stained fibrils with amorphous aggregates (Fig. 4c, h) which were generally smaller in size than that without few-layer Bi_2_Se_3_. In the presence of 300 ng mL^-1^ few-layer Bi_2_Se_3_, primarily positively-stained aggregates with a small size were formed (Fig. 4d). However, Aβ_1-42_ in the presence of 1200 ng mL^-1^ few-layer Bi_2_Se_3_ formed small particles adsorbed on few-layer Bi_2_Se_3_ and no fibrils were observed (Fig. 4e, f, i). The SAED pattern (Fig. 4f insert) indicated that the thin sheet was few-layer Bi_2_Se_3_. The AFM height profiles show that the size of Aβ_1-42_ species decreased with increasing few-layer Bi_2_Se_3_ concentration, which further demonstrates that few-layer Bi_2_Se_3_ inhibits Aβ_1-42_ fibril formation in a dose-dependent manner. The AFM height profile of Aβ_1-42_ in the presence of 60 ng mL^-1^ few-layer Bi_2_Se_3_ was higher than that of Aβ_1-42_ without few-layer Bi_2_Se_3_, which could be attributed to adsorption of Aβ_1-42_ aggregates on few-layer Bi_2_Se_3_ surface. However, the AFM height profile of Aβ_1-42_ in the presence of 1200 ng mL^-1^ few-layer Bi_2_Se_3_ is close to that of bare few-layer Bi_2_Se_3_, which could be attributed to adsorption of Aβ_1-42_ monomers on few-layer Bi_2_Se_3._ TEM images of end-point products of Aβ_1-42_ at 3 h in the presence of few-layer Bi_2_Se_3_ mixture with a thickness 10 ± 8 nm also demonstrated inhibition of Aβ_1-42_ fibril formation by few-layer Bi_2_Se_3_ (Supplementary Fig. S13).

**Figure 5:**
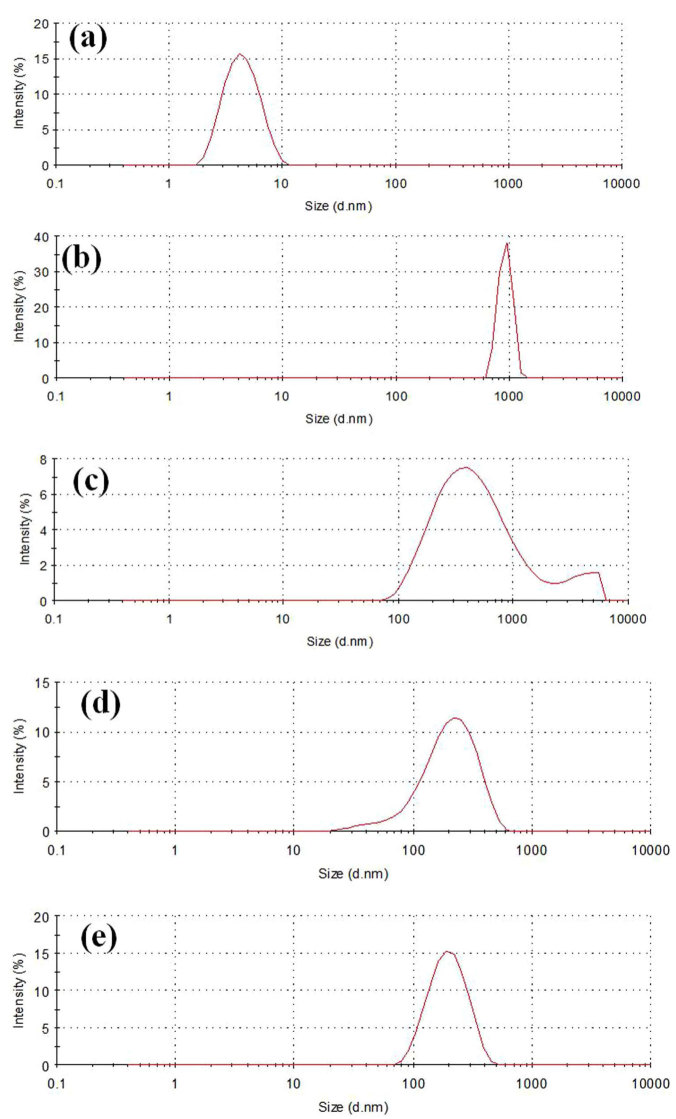
DLS analysis of inhibiting Aβ_1-42_ fibril formation by few-layer Bi_2_Se_3_. (a) Freshly prepared Aβ_1-42_ monomer. (b) Aβ_1-42_ fibril after incubation of Aβ_1-42_ monomer at 37°C for 3 h. (c) Aβ_1-42_ with 12 ng mL^-1^ few-layer Bi_2_Se_3_ after incubation at 37°C for 3 h. (d) Aβ_1-42_ with 1200 ng mL^-1^ few-layer Bi_2_Se_3_ after incubation at 37°C for 3 h. (d) Bare few-layer Bi_2_Se_3_.

We employed dynamic light scattering (DLS) to study the size distribution of particles because DLS can provide a qualitative estimation of the aggregated state of Aβ_1-42_ fibrils (Fig. 5). The freshly prepared Aβ_1-42_ monomer in modified Krebs-Henseliet buffer has a hydrodynamic diameter around 4 nm (Fig. 5a). After incubation of Aβ_1-42_ monomer without few-layer Bi_2_Se_3_ at 37°C for 3 h, large particles with a diameter about 1000 nm were observed. When 12 ng mL^-1^ few-layer Bi_2_Se_3_ was added, the peak at 1000 nm disappeared and broad peaks (400 nm – 4 μm) appeared, indicating parts of the Aβ_1-42_ fibrils remained intact (Fig. 5c). At high concentration of few-layer Bi_2_Se_3_ (1200 ng mL^-1^), no peak at 1000 nm was observed, indicating disappearance of large fibrils (Fig. 5d). The peak is almost the same as that of bare few-layer Bi_2_Se_3_ (Fig. 5e). These results further indicate that few-layer Bi_2_Se_3_ can inhibit Aβ_1-42_ fibril formation.

Furthermore, polyacrylamide gel electrophoresis (PAGE) and cyclic voltammograms (CVs) were employed to confirm the inhibiting effect of few-layer Bi_2_Se_3_ on Aβ_1-42_ fibril formation (Supplementary Fig. S14). Aβ_1-42_ monomer, oligomer and fibril have different molecular weights. Freshly-prepared solution of Aβ_1-42_ monomers displayed a strong band at 5 kDa, the molecular weight of Aβ_1-42_ is 4.5 kDa. After incubation of Aβ_1-42_ at 37°C for 3h, the monomer band (5 kDa) became weak and a band above 100 kDa appeared, indicating the formation of Aβ_1-42_ fibrils. With the addition of few-layer Bi_2_Se_3_ from 12 to 1200 ng mL^-1^, the fibril band became weaker and weaker. When 1200 ng mL^-1^ few-layer Bi_2_Se_3_ was added, the fibril band almost disappeared. To avoid the influence of SDS on Aβ_1-42_ fibril formation, native PAGE was also performed (Supplementary Fig. S14c). After incubation for 3 h, native PAGE indicated that Aβ_1-42_ in the absence of few-layer Bi_2_Se_3_ (lane 2) showed a decreased monomer band compared to freshly prepared Aβ_1-42_ monomer (lane 1). However, when few-layer Bi_2_Se_3_ with different concentrations were added, the monomer band recovered in a dose-dependent manner. The relative quantity of monomer band was calculated from Supplementary Fig. S14c. These results show that few-layer Bi_2_Se_3_ can inhibit Aβ_1-42_ fibril formation in a dose-dependent manner (Supplementary Fig. S14d).

The conductivities of Aβ_1-42_ monomer and fibril on the electrode surface should be different. Therefore, we also investigated the conductivities of glassy carbon electrode (GCE) modified by few-layer Bi_2_Se_3_, Aβ_1-42_ monomer and end-products of Aβ_1-42_ at 3 h with and without few-layer Bi_2_Se_3_ (Supplementary Fig. S14b). GCE modified by few-layer Bi_2_Se_3_ showed good conductivity almost the same as GCE. In the presence of Aβ_1-42_ monomer, there was a small decrease in conductivity. However, a large drop for conductivity was seen when Aβ_1-42_ fibril was introduced, which can be attributed to the insulating property of the fibrils. Interestingly, the conductivity recovered when Aβ_1-42_ with few-layer Bi_2_Se_3_ was introduced under the same concentration, further indicating that few-layer Bi_2_Se_3_ might effectively inhibit Aβ_1-42_ fibril formation, which might be due to the adsorption of Aβ_1-42_ monomers on few-layer Bi_2_Se_3_ to inhibit Aβ fibril formation.

### Inhibition mechanism of Aβ_1-42_ fibril formation by few-layer Bi_2_Se_3_

**Figure 6:**
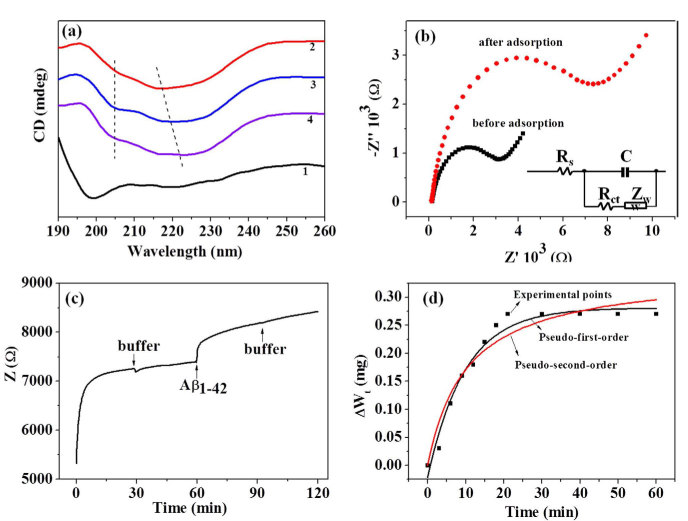
Aβ_1-42_ monomer adsorbing on the surface of few-layer Bi_2_Se_3_. (a) CD spectra of Aβ_1-42_ with and without few-layer Bi_2_Se_3_. (1): Aβ_1-42_ monomer, (2-4): Aβ_1-42_ was incubated at 37°C for 3 h in the presence of few-layer Bi_2_Se_3_ with different concentrations (2: 0, 3: 120 and 4: 2400 ng mL^-1^, respectively). (b) Nyquist diagrams of few-layer Bi_2_Se_3_-modified GCE before and after adsorption of freshly prepared Aβ_1-42_ monomer. Inset: equivalent circuit used to model impedance data in the presence of redox couples. R_s_, electrolyte solution resistance; R_ct_, interfacial electron transfer resistance; C, capacitance of the electric double layer between electrolyte and interface of electrode. The concentrations of few-layer Bi_2_Se_3_ and Aβ_1-42_ monomer were 10 μg mL^-1^ and 10 μM, respectively. (c) Impedance at 10 Hz with time for few-layer Bi_2_Se_3_-modified GCE immersed in modified Krebs-Henseliet buffer. Buffer was added twice at 30 and 90 min, respectively. Aβ_1-42_ monomer was added at 60 min. (d) Weight increment (*Δ*Wt) with time was measured for few-layer Bi_2_Se_3_ coating silicon slice which was immersed in modified Krebs-Henseliet buffer containing 10 μM freshly-prepared Aβ_1-42_ monomers.

In order to investigate the inhibition mechanism, the inhibition process and the interaction of few-layer Bi_2_Se_3_ and Aβ_1-42_ were investigated by circular dichroism (CD), XPS, microbalance, CVs and electrochemical impedance (Fig. 6). In order to investigate whether modification of the secondary structure of Aβ_1-42_ monomer occurred in the presence of few-layer Bi_2_Se_3_, CD spectra were collected (Fig. 6a). Freshly-prepared Aβ_1-42_ monomer displayed a negative peak below 200 nm corresponding to random-coil structure of the peptide. After incubation of Aβ_1-42_ at 37°C for 3 h, Aβ_1-42_ in the absence of few-layer Bi_2_Se_3_ displayed a negative peak at 217 nm corresponding to β-sheet structure of the peptide due to fibril formation. The peak was red-shifted to about 222 nm and a weak peak between 200 and 210 nm appeared with increasing concentration of few-layer Bi_2_Se_3_, which indicated the appearance of α-helix^50^. The percentage of different secondary structure for Aβ_1-42_ monomer and Aβ_1-42_ incubated at 37°C for 3 h in the presence and absence of few-layer Bi_2_Se_3_ was calculated by Jasco secondary structure estimation software (Supplementary Table. S1). These results indicate that few-layer Bi_2_Se_3_ may inhibit Aβ_1-42_ fibril formation by preventing β-sheet structure formation.

In order to confirm that Aβ_1-42_ monomer is adsorbed on the surface of few-layer Bi_2_Se_3_, CVs (Supplementary Fig. S15) and Nyquist diagrams (Fig. 6b) of few-layer Bi_2_Se_3_-modified GCE before and after adsorbing Aβ_1-42_ monomer were collected. Before adsorption, few-layer Bi_2_Se_3_-modified GCE showed good conductivity and the electron transfer resistance (R_ct_) is 2700 Ω. After adsorption, the conductivity decreased and the R_ct_ increased from 2700 to 7800 Ω, indicating that the Aβ_1-42_ monomer had been successfully adsorbed on few-layer Bi_2_Se_3_. To further confirm the results, XPS and EDXA mapping images of few-layer Bi_2_Se_3_ before and after adsorbing Aβ_1-42_ monomer were collected. XPS data demonstrated that the carbon, nitrogen and oxygen contents of few-layer Bi_2_Se_3_ after adsorbing Aβ_1-42_ monomer increased from 48.87%, 5.9% and 21.72% to 54.90%, 10.3% and 30.25%, respectively (Supplementary Fig. S16), indicating adsorption of Aβ_1-42_ monomer on the surface of few-layer Bi_2_Se_3_. EDXA mapping images showed that the Aβ_1-42_ monomer was uniformly adsorbed on the surface of few-layer Bi_2_Se_3_ (Supplementary Fig. S17). These results motivated us to investigate the dynamics of the adsorbing process. First, few-layer Bi_2_Se_3_-modified GCE was prepared and immersed in modified Krebs-Henseliet buffer which buffer and Aβ_1-42_ were added at different times, and impedance spectra with time was collected (Fig. 6c). No obvious impedance change was observed when the buffer was added at 30 and 90 min, while there was a large increase in impedance when Aβ_1-42_ monomer was added at 60 min, which indicated that Aβ_1-42_ monomer gradually adsorbed onto few-layer Bi_2_Se_3_. To quantify the adsorbing amount of Aβ_1-42_ monomer, few-layer Bi_2_Se_3_-coated silicon wafer was hung on a microbalance and the weight was real-time monitored with Aβ_1-42_ monomer adsorbed on the surface of few-layer Bi_2_Se_3_ (Fig. 6d). The weight increased gradually with time and reached equilibrium after 20 min. In order to examine the mechanism and rate-controlling step in the overall adsorption process, pseudo-first-order and pseudo-second-order kinetic models were used to investigate the adsorption process. The nonlinear forms are expressed as the following equations, respectively^51^.
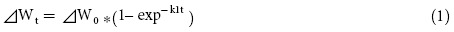

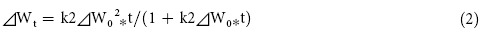
where *Δ*W_0_ and *Δ*W_t_ are the adsorption amounts of Aβ_1-42_ monomer at equilibrium and at time t, respectively. k1 and k2 are the rate constants of pseudo-first-order and pseudo-second-order kinetic equation, respectively.

Figure 6d shows the fitting curves by pseudo-first-order and pseudo-second-order kinetic equations. The fitting of pseudo-first-order kinetic curve overlaps with our experimental data and is better than that of pseudo-second-order kinetic curve. The kinetic parameters of two models are given in Table 2. The value of R^2^ for equation (2) is 0.9343, and the calculated *Δ*W_0,cal_ is far from the experimental value of *Δ*W_0,exp_. The experimental value is consistent with the calculated value from pseudo-first-order kinetic fitting, suggesting the adsorption process can be well-described by the pseudo-first-order kinetic model. The zeta potentials of Aβ_1-42_ monomer and few-layer Bi_2_Se_3_ are -16.3 and -29.4 mV, respectively (Supplementary Fig. S18). Therefore, it is unlikely that the adsorption of Aβ_1-42_ monomer is due to electrostatic interactions. These results suggest that the adsorption rate is mainly controlled by hydrophobic interactions between hemin on the surface of few-layer Bi_2_Se_3_ and Aβ_1-42_.

**Figure 7:**
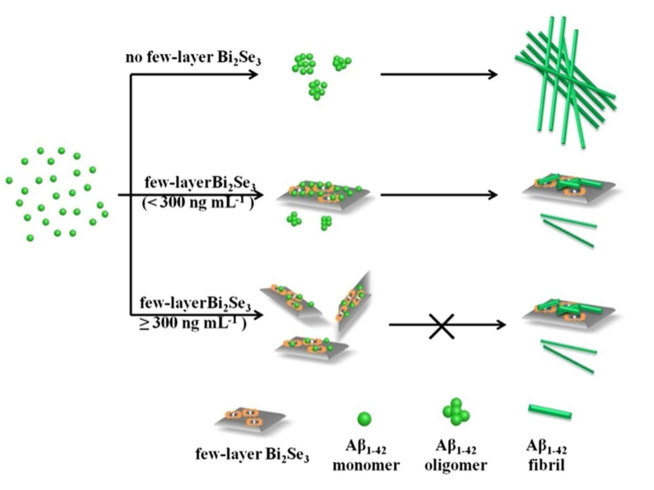
Inhibiting Aβ^1-42^ fibril formation by few-layer Bi_2_Se_3_. Schematic of the mechanism by which few-layer Bi_2_Se_3_ may inhibit Aβ_1-42_ fibril formation.

To represent the suggested mechanism, a simple schematic for Aβ_1-42_ fibril formation with and without few-layer Bi_2_Se_3_ is depicted in Fig. 7. In the absence of few-layer Bi_2_Se_3_, Aβ_1-42_ grows gradually into fibrils. In the ‘nucleation phase’, Aβ monomers with random-coil structure undergo conformational change and aggregate into oligomers. In the ‘elongation phase’, oligomers rapidly grow and form larger aggregates known as fibrils^52^,^53^. When few-layer Bi_2_Se_3_ with a low concentration (<300 ng mL^−1^) is added, Aβ_1-42_ monomers quickly adsorb on the surface of few-layer Bi_2_Se_3_ and grow to form some aggregates due to the relatively high concentration of Aβ_1-42_, while free Aβ_1-42_ monomers in solution aggregate into oligomers, then both Aβ_1-42_ in solution and on few-layer Bi_2_Se_3_ aggregate into fibrils. However, when few-layer Bi_2_Se_3_ with a high concentration (>300 ng mL^−1^) is added, most of Aβ_1-42_ monomers quickly adsorb on the surface of few-layer Bi_2_Se_3_ uniformly. Thus, the amount of free Aβ_1-42_ monomer in solution is low and cannot aggregate to form fibrils.

### Reducing Aβ-mediated peroxidase-like activity and cytoxicity

Recently, heme has been reported to bind Aβ monomer, thus increasing peroxidase-like activity relative to free heme^28^. Therefore, we investigated the effect of few-layer Bi_2_Se_3_ on Aβ-mediated peroxidase-like activity (Supplementary Fig. S19). Hemin-Aβ complexes showed remarkably enhanced peroxidase-like activity relative to free hemin, which is consistent with a study^54^. However, when free hemin is replaced by few-layer Bi_2_Se_3_ containing the same amount of hemin, the enhanced peroxidase-like activity decreased to lower than that of free hemin, but a little higher than that of bare few-layer Bi_2_Se_3_. At the same time, few-layer Bi_2_Se_3_-Aβ complexes incubated at 37°C for 3 h also prevented enhanced peroxidase-like activity. Inhibition of enhanced peroxidase-like activity is attributed to the antioxidant effect of few-layer Bi_2_Se_3_. The results suggest that few-layer Bi_2_Se_3_ could serve as an effective inhibitor of Aβ-mediated peroxidase-like activity.

**Figure 8:**
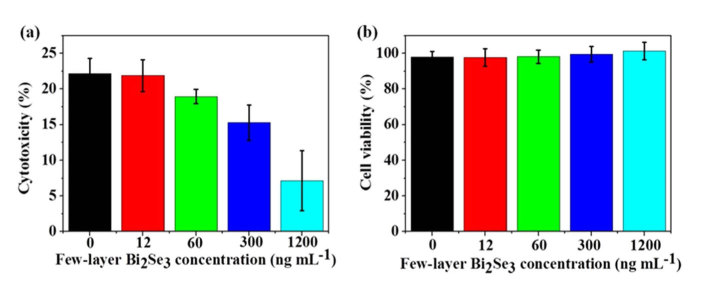
Few-layer Bi_2_Se_3_ reduces the cytoxicity induced by Aβ_1-42_ fibrils for C6 rat glioma cells. Samples were prepared in the presence (a) or absence (b) of Aβ_1-42_. The cytotoxic effect on C6 cells was determined by MTT assay from three separate measurements. Error bars indicate±s.d. Percentage of cytotoxicity was calculated as follows: Cytotoxicity = 100% - cell viability.

To assess the cytoxicity effect of few-layer Bi_2_Se_3_-induced Aβ_1-42_ species, we performed 3-[4,5-Dimethylthiazol-2-yl]-2,5-diphenyltetrazdium bromide (MTT) assays to examine the activity of mitochondrial alcohol dehydrogenase by treating rat glioma cells, C6, with our end-point products at 3 h. Cytoxicity was demonstrated by the reduction of cell viability and the viability is normalized to the one treated with the buffer control. Aβ_1-42_ fibril alone contributed to ~22% cytoxicity to the glioma cells (Fig. 8a). For the end-point products obtained from Aβ_1-42_ monomer incubated with few-layer Bi_2_Se_3_ with different concentrations, the cytoxicity decreased with increasing concentration of few-layer Bi_2_Se_3_ in a dose-dependent manner. When few-layer Bi_2_Se_3_ concentration was 1200 ng mL^-1^, the cytotoxicity was significantly reduced to ~7%. Furthermore, the toxicity of few-layer Bi_2_Se_3_ itself was evaluated by MTT assay (Fig. 8b). Few-layer Bi_2_Se_3_ itself with the concentration used above showed little cell toxicity under our experiment conditions. These results suggest that few-layer Bi_2_Se_3_ can inhibit Aβ_1-42_-induced cell toxicity.

## Discussion

In this paper, we successfully prepared few-layer Bi_2_Se_3_ by liquid-exfoliation with the aid of hemin and found that it could exhibit good performance in inhibiting Aβ fibril formation. There are three reasons that hemin is selected as a stabilizer to exfoliate bulk Bi_2_Se_3_. First, hemin, iron protoporphyrin, is the active center of heme-proteins, such as cytochromes, peroxidase, myoglobin, and hemoglobin that are widely distributed in human body. Thus, hemin is a biocompatible molecule and suitable candidate as a stabilizer for few-layer Bi_2_Se_3_. It also contains a tetrapyrrole macrocycle, and the macrocycle is essentially planar structure, which trends to adsorb on the surface of 2D nanomaterials through π-π stack, hydrophobic and van der Waals interactions^55^. Therefore, hemin benefits the exfoliation of bulk Bi_2_Se_3_ and stabilization of few-layer Bi_2_Se_3_. Second, the macrocycle of hemin also benefits the adsorption of Aβ_1-42_ monomer through π-π stack, hydrophobic and van der Waals interactions because one Aβ_1-42_ monomer contains four aromatic amino acids (three phenylalanines and one tyrosine) and three heterocyclic amino acids (three histidines) (Supplementary Fig. S20). Third, it has been reported that hemin inhibits Aβ aggregation^56^. Our experimental data in Fig. 3e also confirmed the result even though the inhibition efficiency was lower than that of few-layer Bi_2_Se_3_. Therefore, the synergistic effect of hemin and few-layer Bi_2_Se_3_ might enhance the inhibition efficiency. The UV-vis spectra, XRD, SEM, TEM and AFM images of few-layer Bi_2_Se_3_ indicated that few-layer Bi_2_Se_3_ had been successfully prepared. XPS and TGA data show that 11.8% hemin was adsorbed on the surface of few-layer Bi_2_Se_3_. Furthermore, we used fractional centrifugation to obtain few-layer Bi_2_Se_3_ with different sizes and thicknesses, which was confirmed by UV-Vis spectrum, SEM, TEM and AFM images.

Few-layer Bi_2_Se_3_ with different thicknesses were used to evaluate their effect on inhibiting Aβ_1-42_ fibril formation. ThT fluorescence assay was used to probe inhibition efficiency due to its strong fluorescence emission upon binding to cross-β fibrils^47^,^48^,^49^. The inhibition efficiency increased with increasing concentration of few-layer Bi_2_Se_3_. All few-layer Bi_2_Se_3_ with different layers had higher inhibition efficiency than that of hemin, and the inhibition efficiency increased with decreasing layer of few-layer Bi_2_Se_3_, which could be attributed to the adsorption of Aβ monomer on the surface of few-layer Bi_2_Se_3_. The thin few-layer Bi_2_Se_3_ had a larger specific area than that of thick few-layer Bi_2_Se_3_ because specific area increases with decreasing layer of few-layer Bi_2_Se_3_, which benefits adsorption of Aβ_1-42_ monomer. We propose that after adsorption on few-layer Bi_2_Se_3_, the concentration of free Aβ_1-42_ monomer in solution is low and therefore there is a reduction in fibril formation. Aβ_1-42_ monomers, oligomers and fibrils have different morphologies, sizes, molecular weight and conductivities. According to their different morphologies, TEM and AFM images (Fig. 4) support the result that few-layer Bi_2_Se_3_ inhibits Aβ_1-42_ fibril formation. According to their different sizes, DLS has showed that few-layer Bi_2_Se_3_ inhibits Aβ_1-42_ fibril formation (Fig. 5). According to their different molecular weights and conductivities, PAGE and CVs also confirm the inhibition effect of few-layer Bi_2_Se_3_ on Aβ_1-42_ fibril formation.

To understand the inhibition mechanism due to the adsorption of Aβ_1-42_ monomer on few-layer Bi_2_Se_3_, we performed CD, CVs, electrochemical impedance, microbalance, XPS and EDX experiments. CD spectra showed that few-layer Bi_2_Se_3_ prevented the β-sheet structure formation. To further understand this mechanism, CVs, Nyquist diagrams, XPS, EDX mapping before and after adsorption of Aβ_1-42_ monomers were collected. The decreasing conductivity and increasing contents of carbon, nitrogen and oxygen support the result that Aβ_1-42_ monomer was adsorbed onto the surface of few-layer Bi_2_Se_3_. EDX mappings also supported the result that Aβ_1-42_ monomer was uniformly distributed on the surface of few-layer Bi_2_Se_3_. The dynamic adsorptive process was further examined by investigating the impedance change and weight increment with time. Adsorption reached equilibrium at 20 min, and the dynamic adsorptive process was well-described by a pseudo-first-order kinetic model.

Furthermore, few-layer Bi_2_Se_3_ reduced Aβ-mediated peroxidase-like activity and the resulting complex of Aβ_1-42_ and few-layer Bi_2_Se_3_ reduced glial C6 cells toxicities in comparison to those treated with mature Aβ_1-42_ fibrils formed in the absence of few-layer Bi_2_Se_3_. The results suggest that the negatively charged few-layer Bi_2_Se_3_ in the microgram range could potentially serve as an Aβ inhibitor to prevent cell death caused by those detrimental Aβ species.

In summary, we have prepared a new Aβ inhibitor by a simple process. The most important discovery is that few-layer Bi_2_Se_3_ might serve as an Aβ inhibitor and exhibit higher inhibition efficiency against Aβ_1-42_ fibril formation than hemin. The high inhibition efficiency of few-layer Bi_2_Se_3_ may be attributed to its high adsorption capacity for Aβ_1-42_ monomer. Furthermore, few-layer Bi_2_Se_3_, with a good biocompatibility, may reduce enhanced peroxidase-like activity and the cytoxicity induced by Aβ_1-42_. We can envision that few-layer Bi_2_Se_3_ may have the potential for applications in the biomedical field. The metabolism of few-layer Bi_2_Se_3_ *in vivo* and its possible biomedical applications will be investigated in our future work.

## Methods

### Synthesis of bulk Bi_2_Se_3_

Bulk Bi_2_Se_3_ was synthesized as we previously reported^44^. Polyvinyl pyrrolidone (0.9 g) was dissolved in 36 mL of ethylene glycol (EG). Then bismuth oxide powder (1 mmol), selenium powder (3 mmol) and ethylenediamine tetraacetic acid powder (4 mmol) were added into above-mentioned EG. The resulting suspension was stirred vigorously and subsequently sealed in a steel autoclave. The autoclave was heated to 200°C in 30 min and maintained over 20 h for a completed reaction. The as-obtained product was collected by centrifugation at 8000 rpm for 15 min, then washed three times with deionized water and absolute ethanol, and finally dried at 60°C for 96 h in an oven.

### Preparation of few-layer Bi_2_Se_3_

Stock solution of hemin (0.05 mg mL^-1^) was prepared in 0.1% NH_3_·H_2_O aqueous solution. The prepared bulk Bi_2_Se_3_ (100 mg) was dispersed in 200 mL of hemin solution by sonication for 40 h in a sonic bath (KQ-250 DB, 250 W). The resulting dispersion was left to stand for 48 h to allow any unstable aggregates to form. The supernatant was centrifuged at 2000 rpm for 20 min. The precipitate was collected as sample 2000 rpm. After centrifugation, the supernatant was collected and centrifuged at 6000 rpm for 20 min, and the precipitate was collected as sample 6000 rpm. Then, the remaining supernatant was centrifuged at 10000 rpm for 20 min, and the precipitate was collected as sample 10000 rpm. Finally, the collected supernatant was further centrifuged at 13000 rpm for 20 min, and the precipitate was collected as sample 13000 rpm.

### Characterization of bulk Bi_2_Se_3_ and few-layer Bi_2_Se_3_

The micrographs of as-obtained bulk Bi_2_Se_3_ and few-layer Bi_2_Se_3_ were taken using JEOL JEM-2100 TEM (Tokyo, Japan) with an accelerating voltage of 200 kV and LEO-1530 field-emission SEM with an accelerating voltage of 20 kV. The samples were prepared by placing 20 μL of the colloidal solutions on copper grids coating with lacey carbon film for TEM or on small pieces of silicon wafer (P-100) for SEM, and were allowed to dry in air. AFM images were acquired in tapping mode in air using a Digital Instrument Nanoscope®. The samples were prepared by placing 20 μL of 10-fold diluted solutions onto the freshly cleaved mica for 5 min, gently rinsed with deionized water, and dried in vacuum overnight. XRD (Philips PANalytical X’Pert) equipped with Cu Ka radiation (λ = 1.542 Å) over the 2θ range of 10–80° was used to characterize the structure of few-layer Bi_2_Se_3_. Sample was prepared by depositing a film on the surface of glass slid. The UV–vis absorption spectra were measured on UV–vis spectrometer (UV-2550, Shimadzu), equipped with 1 cm quartz cells. The spectra were recorded in the wavelength range of 200–800 nm at a scan speed of 400 nm min^-1^.

### Preparation of Aβ monomer solution

The Aβ_1−42_ monomer stock solution (200 μM) was prepared according to the previous reports^8^. Briefly, 1 mg lyophilized Aβ_1−42_ (purity: 98.3%, measured by HPLC; molecule weight: 4514 Da, measured by mass spectrometry) synthesized by GL Biochem Ltd. (Shanghai, China) was dissolved in freshly prepared 942 μL of NaOH (10 mM) solution by sonication for 5 min under 100 W at ice cold condition. The peptide was fully dissolved under alkaline condition and existed only as monomer^57^. Peptide stock was stable in this form for 24 h at 4°C.

### Effect of few-layer Bi_2_Se_3_ on Aβ_1−42_ fibril formation

The previously prepared Aβ_1-42_ monomer solution was mixed with designed volumes of few-layer Bi_2_Se_3_ (6 μg mL^-1^) solution and then diluted by sterilized modified Krebs-Henseliet buffer to the final solution of 10 μM Aβ_1-42_ with 0, 12, 60, 300 and 1200 ng mL^-1^ few-layer Bi_2_Se_3_, respectively. The samples were incubated at 37°C in 1.5 mL of Eppendorf tubes from 0 to 3 hours, and some of samples were taken out at the desired incubation time and used for the following measurements of ThT fluorescence, TEM, AFM, DLS, SDS-PAGE, CD, CVs, Nyquist diagrams and MTT assay. The physiologically-significant medium was a modified Krebs-Henseliet buffer including 118.5 mM sodium chloride, 4.8 mM potassium chloride, 1.2 mM magnesium sulfate, 1.4 mM calcium chloride, 11.0 mM glucose and it was buffered at pH 7.40 ± 0.05 with 100 mM Piperazine-1,4-bis(2-ethanesulfonic acid)^58^. The buffer also included 0.05% w/v sodium azide as an antimicrobial. All treatments were prepared and maintained at 37°C to accurately reflect physiological milieu.

### ThT fluorescence assay

The fibril formation of Aβ_1-42_ with or without inhibitors was evaluated by ThT fluorescence with a fluorescence spectrophotometer (FLS-920, Edinburgh Instruments). 50 μL of Aβ_1−42_ (200 μM) was mixed with few-layer Bi_2_Se_3_ with different layers of required concentration and incubated in a 1.5 mL of sterile brown centrifuge tube, which included 10 μM ThT and 0.05% sodium azide in the modified Krebs-Henseliet buffer. The final volume was 1 mL. The Aβ concentration was fixed at 10 μM (45.14 μg mL^-1^) and concentration of few-layer Bi_2_Se_3_ was changed from 0 to 1200 ng mL^-1^. The samples were loaded on a thermomixer (Eppendorf, Germany) at 37°C for 0–3 h during which samples were collected and measured. Three replicates were performed and averaged.

### TEM and AFM measurements

Final solution of 10 μM Aβ_1-42_ with 0, 12, 60, 300 and 1200 ng mL^-1^ few-layer Bi_2_Se_3_ incubated at 37°C for 3 h were used for TEM and AFM measurements. The morphologies of Aβ_1−42_ with and without few-layer Bi_2_Se_3_ were confirmed by TEM and AFM. For TEM measurements, samples were deposited on 400-mesh Formvar carbon-coated copper grids for 5 min. Negative staining was performed by using 2% uranyl acetate for 5 min and the grids were rinsed once with double distilled water. The samples were examined with a JEOL JEM-2100 TEM with an accelerating voltage of 200 kV. For AFM measurements, 10 μL of samples were freshly prepared and swiftly diluted 10-fold in deionized water. 10 μL of the diluted sample was mounted onto the freshly cleaved mica for 5 min, gently rinsed with deionized water, and dried in vacuum overnight. Images were acquired under atmosphere in a tapping mode.

### DLS

Samples of 20 μM Aβ_1-42_ monomer, 20 μM Aβ_1-42_ incubated at 37°C for 3 h with 0, 24 and 2400 ng mL^-1^ few-layer Bi_2_Se_3_, and 6 μg mL^-1^ few-layer Bi_2_Se_3_ were used for DLS measurements. The samples were 2-fold diluted in double distilled water and subjected to size analysis by Zetasizer Nano (Malvern Instruments, Worchestershire, UK) using disposable solvent resistant micro cuvette (ZEN0040) at room temperature.

### SDS-PAGE and Native-PAGE analysis

20 μM Aβ_1-42_ monomer, 20 μM Aβ_1-42_ incubated at 3 h with 0, 24, 120, 600 and 2400 ng mL^-1^ few-layer Bi_2_Se_3_ were used for SDS or Native-PAGE analysis. For SDS-PAGE analysis, the samples (10 μL) were mixed with 2×PAGE sample loading buffer (10 μL). Then samples were run on 12% PAGE gel at 80 V for 0.5 h followed by 120 V for 1.5 h. The gel was stained by coomassie blue. For Native-PAGE analysis, samples were run on 12% Tris/glycine gel at 100 V for 10 min followed by 200 V for 0.5 h. The gel was stained using silver stain and the relative quantity was estimated using Bio-Rad’s Image Lab 4.1 software. The bands were both visualized using gel imaging and analysis system (Bio-rad Gel Doc XR).

### CD spectra

20 μM Aβ_1-42_ monomer, 20 μM Aβ_1-42_ incubated at 3 h with and without 0, 120, and 2400 ng mL^-1^ few-layer Bi_2_Se_3_ were used for CD spectra. The CD spectra were measured from 190 to 260 nm at room temperature on a Jasco J-810 spectrometer (Tokyo, Japan) using a cell with a path length of 0.1 cm. Data were collected every 0.2 nm with 3 nm bandwidth at a scan speed of 50 nm min^-1^ and response time of 4 seconds. All spectra were collected in a triplicate and a background-corrected against buffer blank.

### Inhibiting Aβ_1-42_ fibril formation by CVs

10 μM of Aβ_1-42_ monomer, 10 μM Aβ_1-42_ incubated at 37°C for 3 h with 0, and 1200 ng mL^-1^ few-layer Bi_2_Se_3_, and 1200 ng mL^-1^ few-layer Bi_2_Se_3_ were used for CVs measurements. The glass-carbon electrode (GCE, Φ = 3 mm) was polished with 0.3 and 0.05 μm alumina slurry, rinsed thoroughly with doubly distilled water between each polishing step, then GCE was washed successively with 1:1 nitric acid, ethanol, and doubly distilled water in an ultrasonic bath and dried in air. 10 μL of samples were dropped onto the GCE to prepare modified electrodes. CVs measurements were performed on an electrochemical workstation (CHI660C, CH Instrument, USA). The three-electrode system consisted of a platinum wire as auxiliary electrode and an Ag/AgCl (saturated KCl) as reference. Working electrodes were GCEs modified with samples. CV measurements were performed in 6.0 mM K_3_[Fe(CN)_6_] and 1.0 M KCl solution with the scan rate of 100 mV s^-1^.

### Adsorption by CVs and impedance measurements

10 μL of few-layer Bi_2_Se_3_ (10 μg mL^-1^) was dropped onto the GCE to prepare few-layer Bi_2_Se_3_-modified electrode. For adsorbing Aβ_1-42_ monomer, few-layer Bi_2_Se_3_-modified electrode was immersed in modified Krebs-Henseliet buffer containing 10 μM Aβ_1-42_ monomer for 10 min, and then immersed in modified Krebs-Henseliet buffer for 30 seconds to remove free Aβ_1-42_ monomers. Few-layer Bi_2_Se_3_-modified electrode before and after adsorbing Aβ_1-42_ monomer were used for CVs and impedance measurements. CVs were performed almost the same as above. The impedance measurements were performed in 5.0 mM [Fe(CN)_6_]^3-/4-^ and 1.0 M KCl solution. The AC voltage amplitude was 5 mV and the voltage frequencies ranged from 0.1 Hz to 10^5^ Hz.

### Adsorption kinetics process by impedance measurements and real-time weight measurements

The impedance measurement with time was performed in modified Krebs-Henseliet buffer at 10 Hz. Few-layer Bi_2_Se_3_-modified electrode was subjected to successive incubations first in modified Krebs-Henseliet buffer without Aβ_1-42_ as a pre-equilibration step. Buffer was added twice at 30 min and 90 min, respectively. Aβ_1-42_ monomer was added at 60 min.

Real-time weight measurement was performed on a microbalance (KSV NIMA). Silicon wafer was washed successively with 1:1 nitric acid, ethanol, and doubly distilled water in an ultrasonic bath and dried in air. 50 μL of few-layer Bi_2_Se_3_ (10 μg mL^-1^) dispersion was dropped onto silicon wafer to prepare few-layer Bi_2_Se_3_-coated silicon wafer. Then the modified silicon wafer was dried naturally overnight. Then few-layer Bi_2_Se_3_-coated silicon wafer was hung on a microbalance and immersed in modified Krebs-Henseliet buffer containing 10 μM Aβ_1-42_ monomers. Thus, the weight of adsorbed Aβ_1-42_ monomers was *in situ* monitored.

### Inhibition of Aβ-mediated peroxidase-like activity

The inhibition assay of Aβ-mediated peroxidase-like activity was performed as follows. Firstly, 12 μg mL^-1^ of few-layer Bi_2_Se_3_ or 1.416 μg mL^-1^ of hemin in the presence and absence of 50 μM Aβ_1-42_ monomers were incubated at 37°C for 0 h or 3 h in a 1.5 mL of sterile centrifuge tube and served as catalyst for peroxidase-like activity assay. Kinetic measurements were carried out in time course mode by monitoring the absorbance change at 652 nm on a TU-1901 UV-vis spectrophotometer. Peroxidase-like activity experiments were performed using the above samples as catalyst in a reaction volume of 600 μL acetic acid-sodium acetate buffer solution (0.02 M, pH 4, 25°C) with 100 μM 3,3′,5,5′-Tetramethylbenzidine as substrate and 10 mM hydrogen peroxide.

### Inhibition of Aβ-mediated cytotoxicity

The rabbit glial C6 cells (from cell storeroom of Chinese Academy of Sciences) were incubated at 37°C under 5% CO_2_, and were cultured in DMEM media (Gibco, Invitrogen, Carlsbad, CA, USA) with 10% fetal bovine serum (FBS) (Biological Industry, Kibbutz Beit-Haemek, Israel) in a humid camber. A total of 1×10^5^ cells were seeded overnight in the growth medium in a polystyrene 96-well plate (Corning, NY, USA). The growth medium was then discarded and the cells were washed twice by 1×phosphate buffered saline (PBS). 1×PBS was prepared by dissolving 0.24 g KH_2_PO_4_, 1.44 g Na_2_HPO_4_, 0.2 g KCl and 8.0 g NaCl into 1 L of water. pH was adjusted to 7.4 with HCl. The final concentration was 10 mM phosphate, 137 mM NaCl and 2.7 mM KCl. Then, the FBS-free media (50 μL) was added into each well. Next, the cells were treated with 50 μL of the end-point products as described in text. The cells were incubated for additional 24 h in the growth chamber and then 50 μL of MTT (5 mg mL^-1^ in DMEM without FBS) was added into each well and incubated for another 4 h. The media was discarded and dimethylsulfoxide was used to lyze the cells until the purple crystals were fully dissolved. Absorbance at 570 nm was measured by a microplate reader (SpectraMax M5, Molecule Devices). Three replicates were performed and the data were averaged (n = 3). Background signals from sample treatment without cells were subtracted. Each data set was normalized using the reading obtained from the buffer controls and the cytotoxicity data were obtained by subtracting the viability data from 100%.

## Additional Information

**How to cite this article**: Peng, J. *et al*. Few-layer bismuth selenides exfoliated by hemin inhibit amyloid-ß_1-42_ fibril formation. *Sci. Rep.* **5**, 10171; doi: 10.1038/srep10171 (2015).

## Supplementary Material

Supplementary Information Supplementary Figures 1-3 and Supplementary Tables 1-3

## Figures and Tables

**Table 1:** Size, thickness of few-layer Bi_2_Se_3_ at differently centrifugal speeds and their inhibition efficiency towards Aβ.

**Inhibitor**	Hemin	**Mixture**	**2000 rpm**	**6000 rpm**	**10000 rpm**	**13000 rpm**
Size (nm) a	/	447 ± 373	637 ± 183	343 ± 73	243 ± 53	105 ± 31
Thickness (nm) b	/	10 ± 8	40 ± 10	15 ± 3	6 ± 2	3 ± 1
Inhibition efficiency (%) c	19.7	44.7	/d	33.7	51.7	67.4

(a) The size was obtained from TEM images. Mean and standard deviation are given, n = 10.

(b) The thickness was obtained from AFM images. Mean and standard deviation are given, n = 10.

(c) The inhibition efficiency was analyzed by ThT fluorescence assay. It is calculated as follows: Inhibition efficiency = (F_0_-F_i_)/F_0_ × 100%, F_i_ and F_0_ represent the ThT fluorescence intensity of Aβ_1-42_ with and without inhibitor, respectively. Aβ_1-42_ and inhibitor were incubated at 37°C for 3 h as showed in Fig.3.

(d) Few-layer Bi_2_Se_3_ handled at 2000 rpm was thought as bulk Bi_2_Se_3_ which has not been exfoliated as expected. The dispersity of bulk Bi_2_Se_3_ in aqueous solution is poor.

**Table 2:** Kinetic constants of the pseudo-first-order and pseudo-second-order kinetic models.

	**Pseudo-first-order model**			**Pseudo-second-order model**		
*Δ*W_0,exp_ (mg)	*Δ*W_0,cal_ (mg)	k1 (min^−1^)	R^2^	*Δ*W_0,cal_ (mg)	k2 (mg^−1^ min^−1^)	R^2^
0.27	0.28	0.10	0.9698	0.35	0.28	0.9343
