# Supplementary material for: Few-layer bismuth selenides exfoliated by hemin inhibit amyloid-β1–42 fibril formation
Source: Sci Rep. 2015 May 28;5:10171. doi: 10.1038/srep10171 (PMC4446900; doi:10.1038/srep10171)
Supplement: Supplementary Information — Supplementary Figures 1-3 and Supplementary Tables 1-3 [file srep10171-s1.pdf]

Supplementary information

Few-layer bismuth selenides exfoliated by hemin inhibit amyloid-β_{1-42} fibril formation

Jian Peng [1, 2], Yunjing Xiong[2], Zhiqin Lin[2], Liping Sun[2]* & Jian Weng[2, 3]*

[1]College of Chemistry and Chemical Engineering, Xiamen University, Xiamen 361005, P.R. China.

[2] College of Materials, Xiamen University, Xiamen 361005, P.R. China.

[3]ShenZhen Research Institute of Xiamen University, Shenzhen 518057, China

Correspondence and requests for materials should be addressed to L.S. (email: sunliping@xmu.edu.cn) or J.W. (email: jweng@xmu.edu.cn)

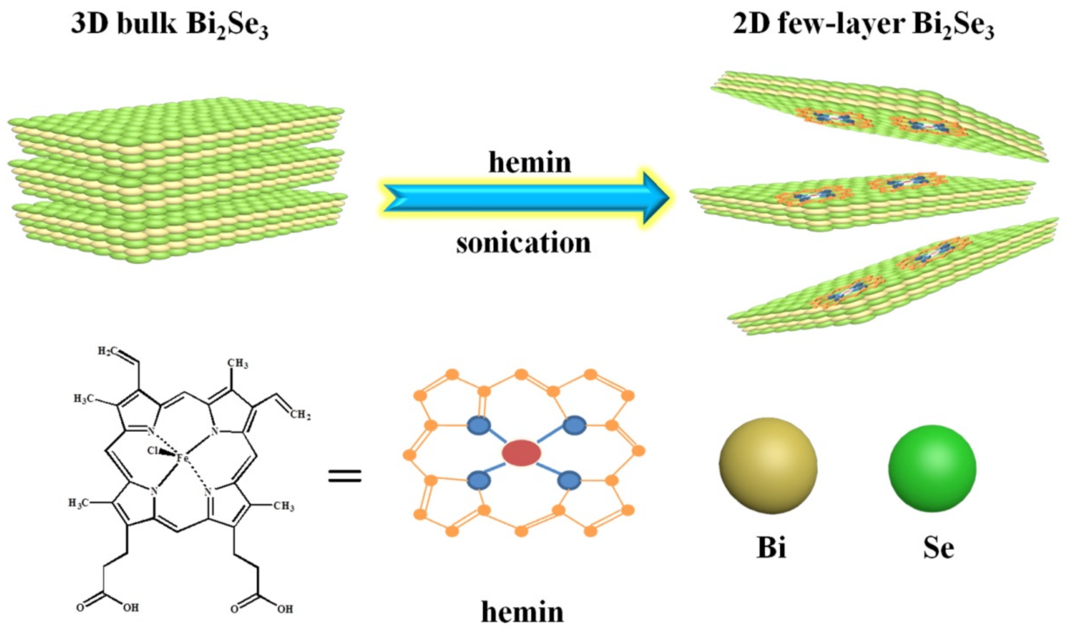

Supplementary Figure S1 | Schematic of liquid-phase exfoliation. The layered Bi_2Se_3 crystal is sonicated in hemin aqueous solution, giving rise to exfoliation and nanosheets formation. Hemin as a small molecule can disperse nanosheets by adsorption on their surface, and then stabilize few-layer Bi_2Se_3.

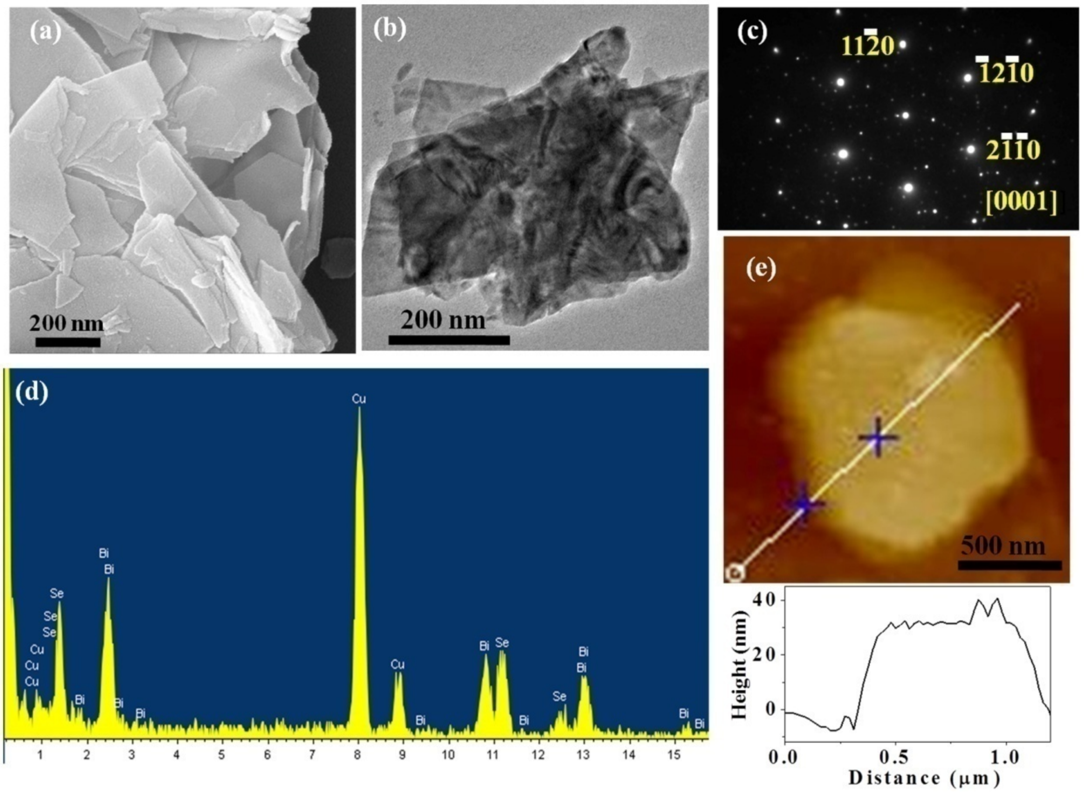

Supplementary Figure S2│3D structure of as-prepared bulk Bi_2Se_3. (a) SEM image of bulk Bi_2Se_3. (b) TEM image of bulk Bi_2Se_3. (c) SAED pattern of bulk Bi_2Se_3. (d) EDX spectrum of bulk Bi_2Se_3. The atomic ratio of Bi and Se is 1:1.47, indicating an approximate A_2B_3 type compound. (e) AFM image and the corresponding height profile of bulk Bi_2Se_3. The thickness of bulk Bi_2Se_3 is about 40-50 nm.

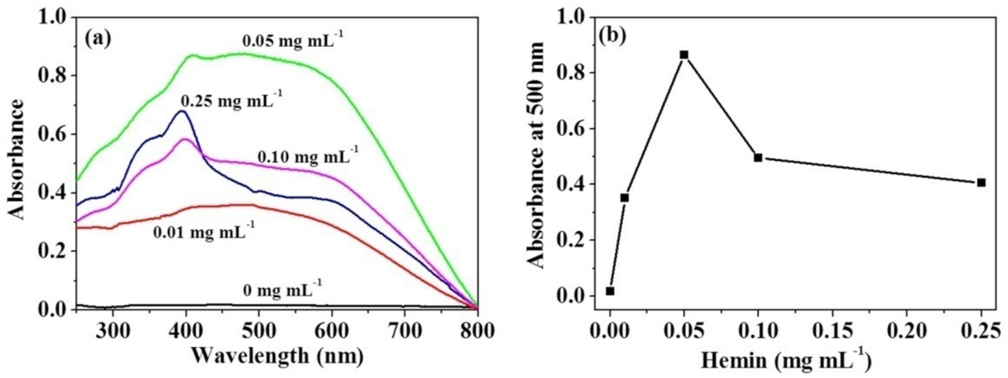

Supplementary Figure S3 | Effect of hemin concentration on exfoliation of bulk Bi_2Se_3. (a) UV-vis absorption spectra of few-layer Bi_2Se_3 after 40 h of sonication in the presence of hemin with different concentrations. (b) Absorption at 500 nm of as−obtained few−layer Bi_2Se_3 dispersion prepared in the presence of hemin with different concentrations.

A series of few-layer Bi_2Se_3 dispersion solutions were prepared by adding same amount of powdered bulk Bi_2Se_3 to different concentrations of hemin dissolved in 0.1% ammonia water ($NH_3{\cdot}H_2O$). Fig. S3 demonstrates that both low or high concentrations of hemin would decrease the yield of few-layer Bi_2Se_3. When the concentrtion of hemin was low, some few-layer Bi_2Se_3 exfoliated from bulk Bi_2Se_3 could not be protected by hemin and aggregated with each other again. On the contrary, at high concentration of hemin, bulk Bi_2Se_3 was wrapped up completely by the excess hemin, which would weaken the effect of sonication on bulk Bi_2Se_3 and prevented the Bi_2Se_3 sheets to be exfoliated from bulk Bi_2Se_3 stacking during sonication process. In our experiment, the optimal concentration of hemin was 0.05 mg mL^{-1}.

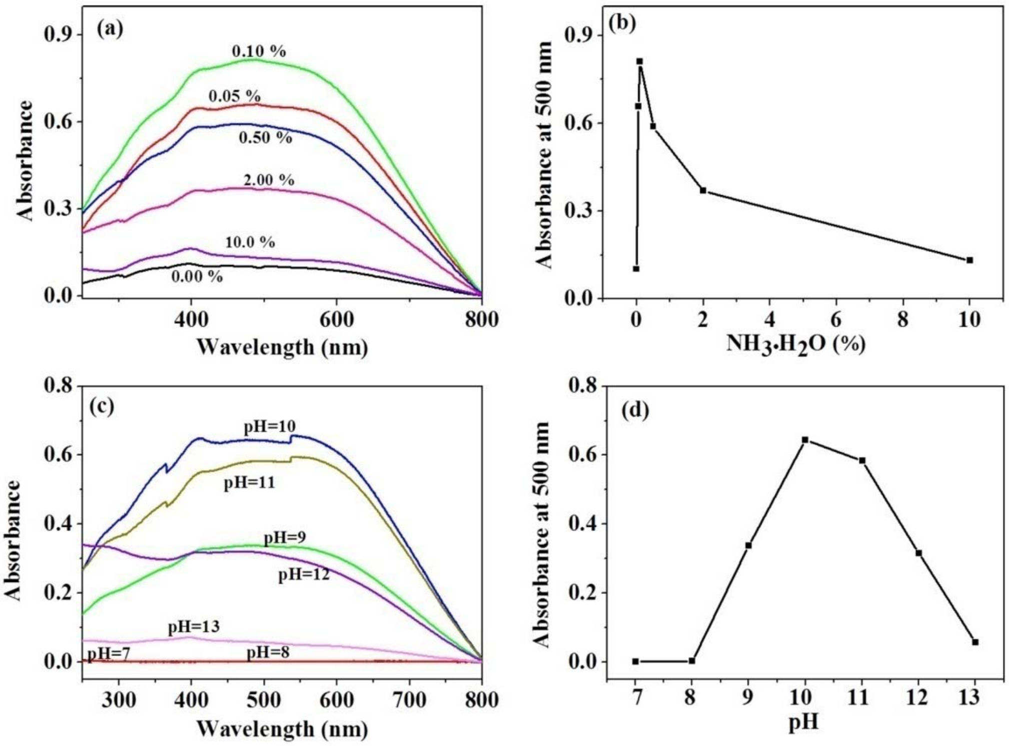

Supplementary Figure S4│Effect of $NH_3{\cdot}H_2O$ concentration and pH on exfoliation of bulk Bi_2Se_3. (a) UV-vis absorption spectra of few-layer Bi_2Se_3 after 40 h of sonication with 0.05 mg mL^{-1} hemin in $NH_3{\cdot}H_2O$ with different concentrations. (b) Absorption at 500 nm of as−obtained few−layer Bi_2Se_3 dispersion solution prepared in $NH_3{\cdot}H_2O$ with different concentrations. (c) UV-vis absorption spectra of few-layer Bi_2Se_3 after 40 h of sonication in the presence of 0.05 mg mL^{-1} hemin with different pH values adjusted by 0.1 M NaOH solution. (d) Absorption at 500 nm of as−obtained few−layer Bi_2Se_3 dispersion solution prepared at different pH values.

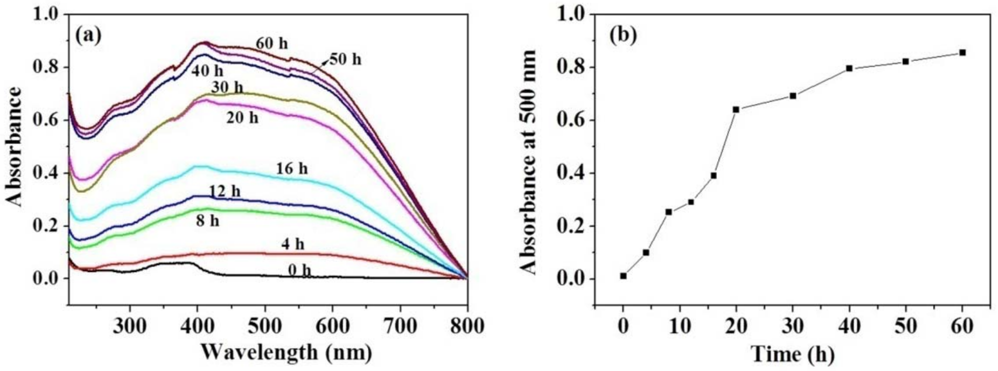

Supplementary Figure S5 | Effect of sonication time on exfoliation of bulk Bi_2Se_3. (a) UV-vis absorption spectra of few-layer Bi_2Se_3 after sonication with different times in the presence of 0.05 mg mL^{-1} hemin and 0.1% $NH_3 \cdot H_2O$. (b) Absorption at 500 nm of as-obtained few-layer Bi_2Se_3 dispersion solution prepared after different sonication times.

A series of few-layer Bi_2Se_3 dispersion solutions were prepared by adding powdered bulk Bi_2Se_3 to 0.05 mg mL^{-1} hemin solution. Then these dispersion solutions were sonicated for various times from 0 to 60 h. Fig. S5 presents the absorption curves of few-layer Bi_2Se_3 suspension after different sonication times. The result demonstrates that the absorbance at 500 nm increases gradually with extending ultrasonic time, which indicates that more few-layer Bi_2Se_3 can be received with ultrasonic time increasing. The yield of few-layer Bi_2Se_3 increased a little after 40 h. Therefore, the optimally ultrasonic time was 40 h.

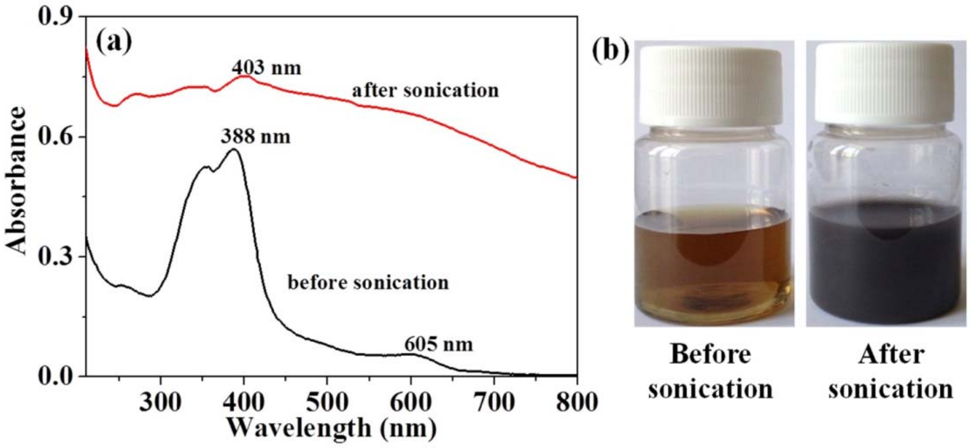

Supplementary Figure S6 | UV-vis absorption spectra and optical photograph of the mixture. (a) UV-vis absorption spectra of the mixed solution of bulk Bi_2Se_3 and hemin before and after sonication. (b) Optical photograph of the mixed solution before and after sonication.

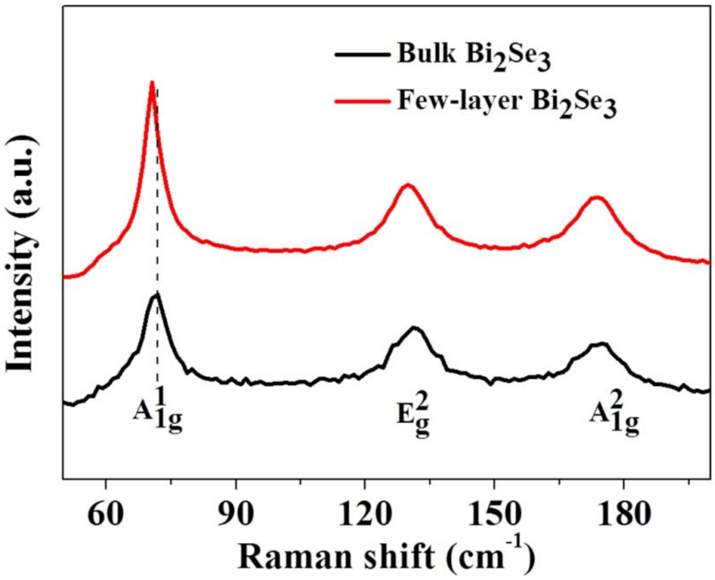

Supplementary Figure S7│Raman spectra of bulk Bi_2Se_3 and few-layer Bi_2Se_3. The peaks of bulk Bi_2Se_3 at ~72, ~131, and~174 cm^{-1}, are assigned to A^1_{1g}, E^2_g and A^2_{1g} vibrational modes, respectively. The dashed vertical line indicates a redshift of A^1_{1g} mode in few-layer Bi_2Se_3 compared with that of bulk Bi_2Se_3.

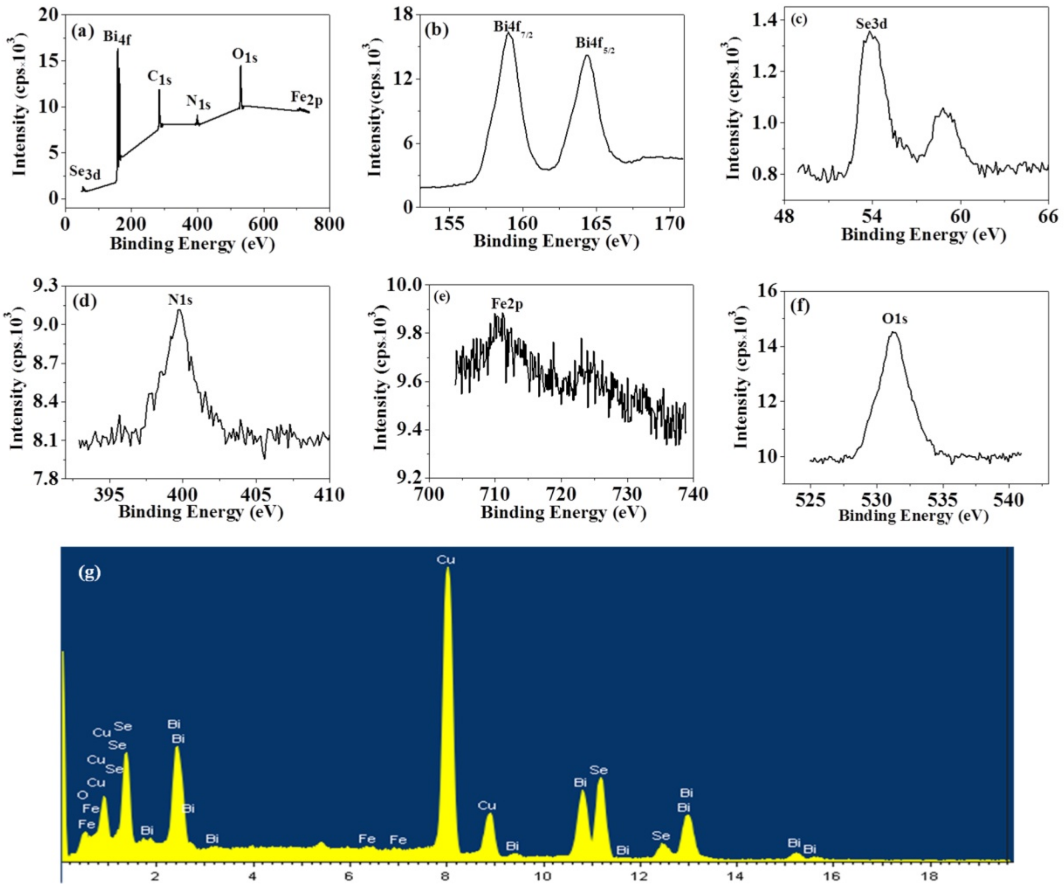

Supplementary Figure S8│X-ray photoelectron spectroscopy (XPS) and EDX spectrum of few-layer Bi_2Se_3. (a) Wide-scan spectrum. (b) High-resolution at bismuth region (Bi4f). (c) High-resolution at selenium region (Se3d). (d) High-resolution at nitrogen region (N1s). (e) High-resolution at iron region (Fe2p). (f) High-resolution at oxygen region (O1s). (g) EDX spectrum of few-layer Bi_2Se_3.

According to the XPS spectra (Fig. S8), the content of iron element (Fe) in few-layer Bi_2Se_3 is 1.03%. However, hemin (molecular formula: $C_{34}H_{32}ClFeN_4O_4$, Mw=651.94 g mol^{-1}) contains 8.59% Fe. Thus, the calculated content of hemin by XPS is 12.0%, which is almost same to that calculated by TGA curves in Fig. 1g.

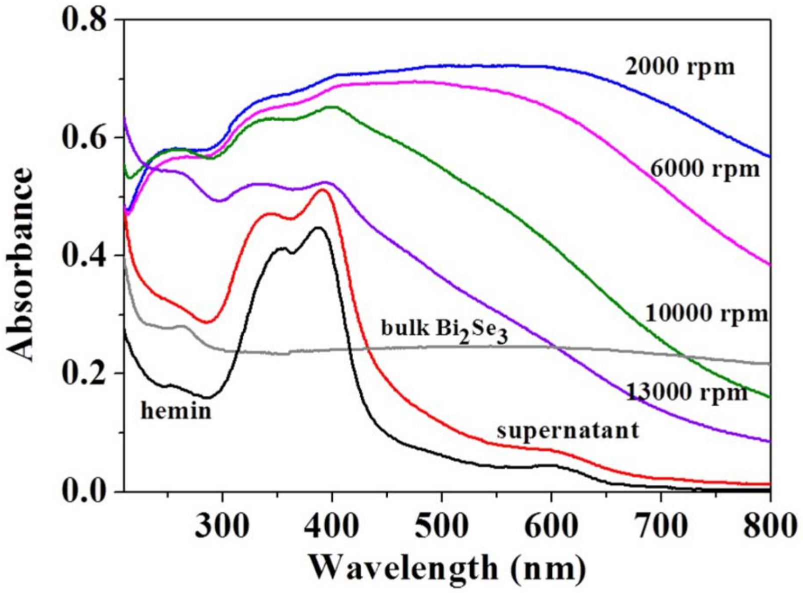

Supplementary Figure S9│UV-vis absorption spectra of hemin, bulk Bi_2Se_3, precipitate and supernatant of few-layer Bi_2Se_3 stock solution centrifuged at 2000, 6000, 10000 and 13000 rpm, respectively.

The optical absorption spectra of few-layer Bi_2Se_3 in solution exhibit a strong absorption band in the visible light region, which is different from bulk Bi_2Se_3 without any absorption peak in this region, resulting from quantum size effect. Therefore, the optical absorption of few-layer Bi_2Se_3 is size-dependent.

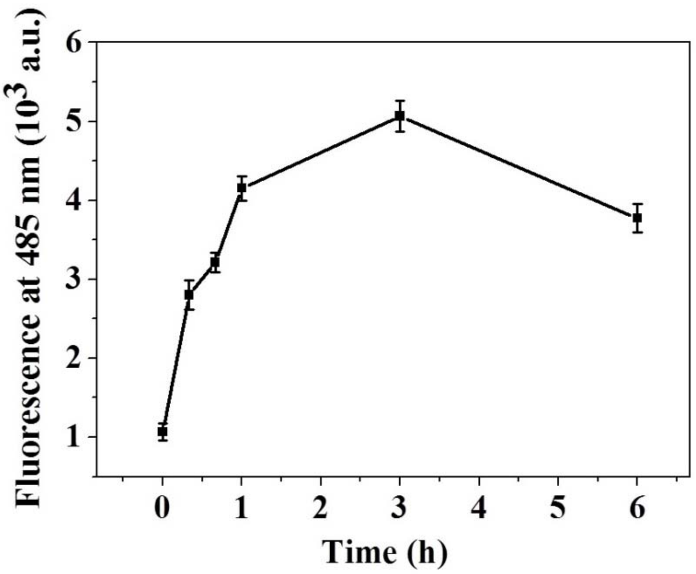

Supplementary Figure S10 | Kinetics curve of A$\beta_{1\text{-}42}$ fibril formation in modified Krebs-Henseliet buffer**.** Time-dependent ThT fluorescence was monitored at 485 nm with an excitation of 442 nm. Three replicates were performed.

The ThT fluorescence intensity increases with increasing time due to the formation of Aβ fibril, and then declines after 3 h. Our explanation for the latter involves accessibility of ThT binding sites on the rapidly aggregating peptide. The aggregating peptide will precipitate with time extending.

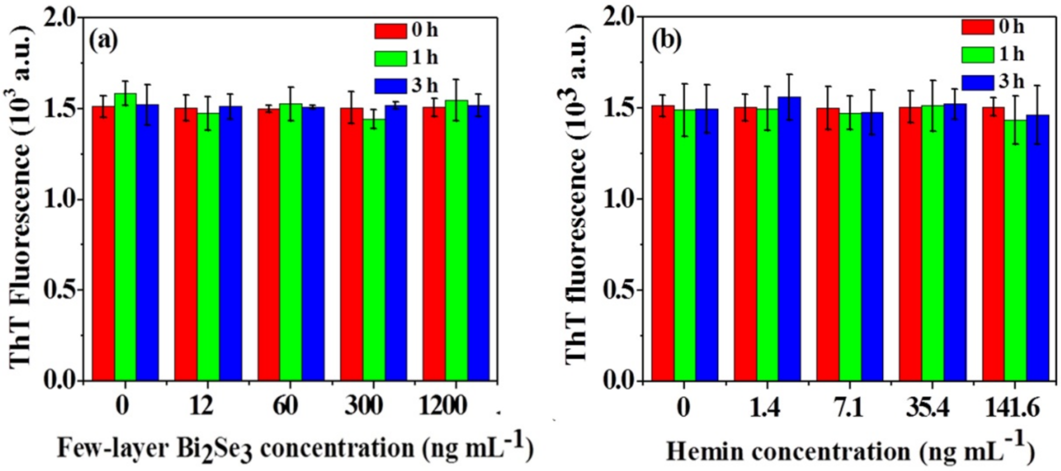

Supplementary Figure S11 | ThT fluorescence intensities versus few-layer Bi_2Se_3 (a) and hemin (b) concentrations at 0, 1 and 3 h in the absence of $A\beta_{1\text{-}42}$ monomer. Three replicates were performed.

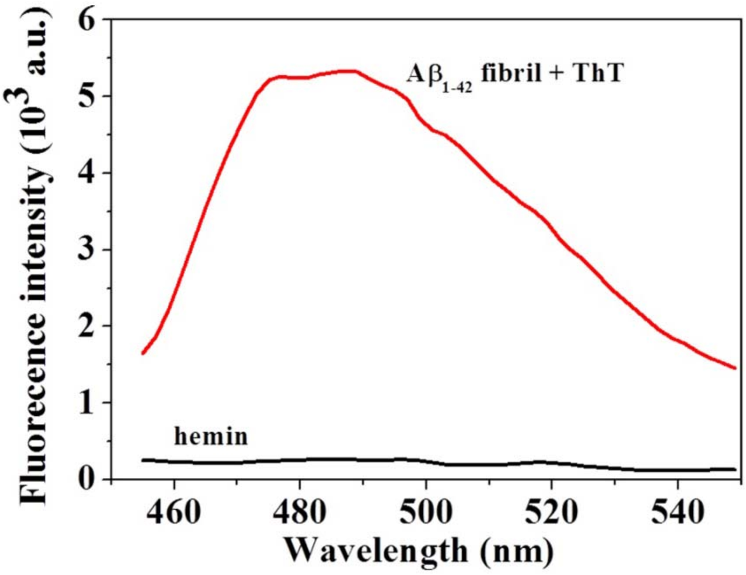

Supplementary Figure S12 | ThT fluorescence spectrum of $A\beta_{1\text{-}42}$ fibril (red line) and intrinsic fluorescence spectrum (black line) of hemin. The excitation wavelength is 442 nm.

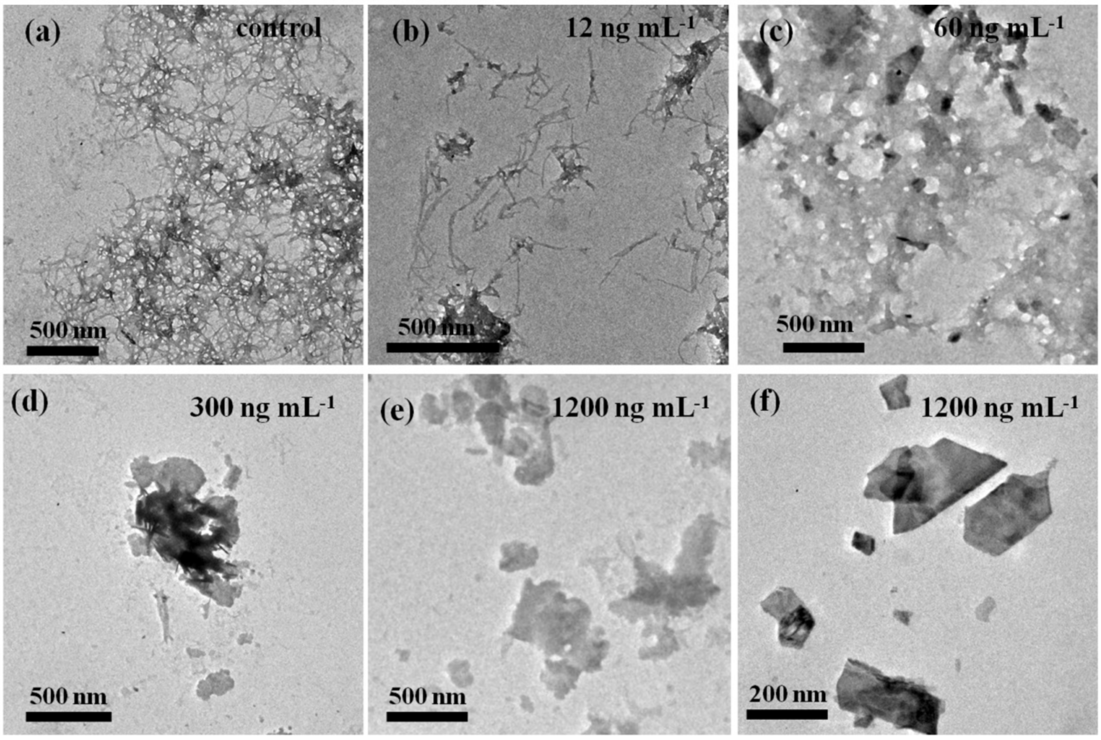

Supplementary Figure S13 | TEM images of the A$\beta_{1\text{-}42}$ species with and without few-layer Bi_2Se_3 mixture (10 ± 8 nm) without fractional centrifugation monitored by TEM. The concentration of few-layer Bi_2Se_3 and scale bars are indicated in each image.

In the absence of few-layer Bi_2Se_3, A$\beta_{1\text{-}42}$ formed long, smooth, and entangled fibrils as expected (Fig. S13a). However, after incubation of A$\beta_{1\text{-}42}$ with different concentrations of few-layer Bi_2Se_3 mixture without fractional centrifugation, different Aβ species were observed. With increasing concentration of few-layer Bi_2Se_3, the fibril was decreased and completely inhibited. The result further demonstrates that few-layer Bi_2Se_3 would inhibit A$\beta_{1\text{-}42}$ fibril formation.

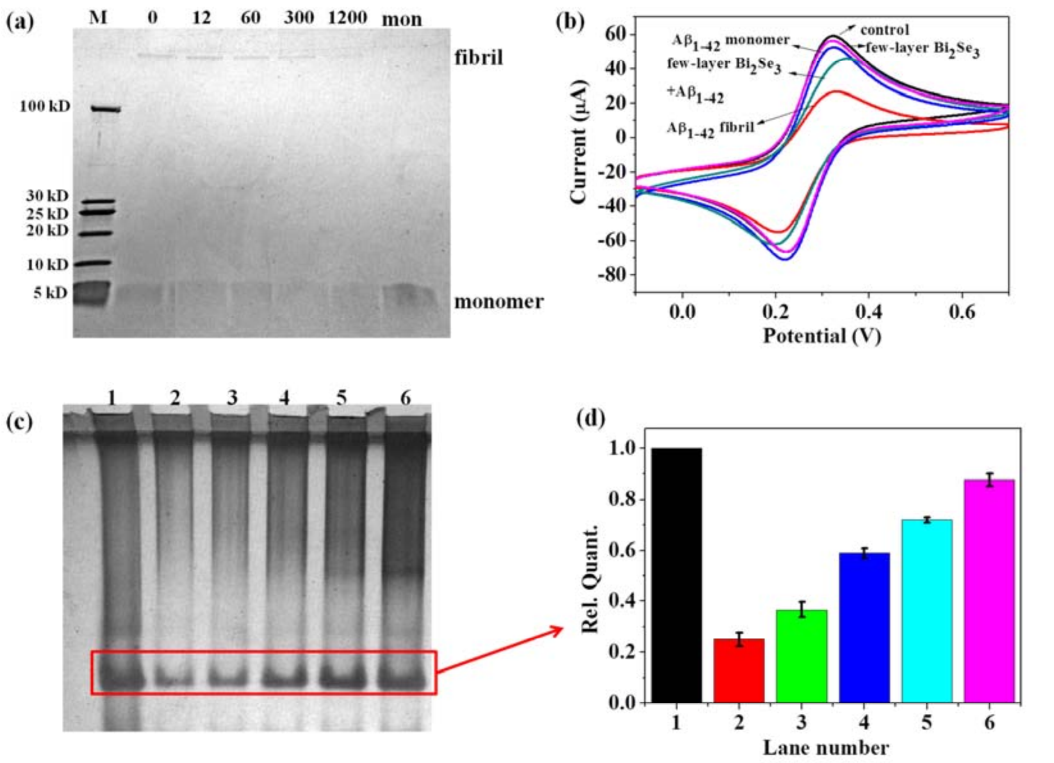

Supplementary Figure S14│Few-layer Bi_2Se_3 Inhibits $A\beta_{1-42}$ fibrillation monitored by PAGE and CVs. (a) SDS-PAGE photograph of $A\beta_{1-42}$ monomer (mon) and $A\beta_{1-42}$ incubated for 3 h in the presence of few-layer Bi_2Se_3 with different concentrations from 0 to 1200 ng mL^{-1} (0-1200). Marker (M) was given. $A\beta_{1-42}$ monomer and fibril band were indicated, respectively. The gel was stained by coomassie blue. (b) CVs of bare GCE (control) and GCEs modified with few-layer Bi_2Se_3 (1200 ng mL^{-1}), $A\beta_{1-42}$ monomer and $A\beta_{1-42}$ incubated for 3 h at 37℃ with 1200 ng mL^{-1} few-layer Bi_2Se_3. The CVs measurements were carried out in 6.0 mM $K_3[Fe(CN)_6]$ and 1.0 M KCl solution. (c) Native PAGE with silver stain. Lane 1: control 1 ($A\beta_{1-42}$ monomer only), lane 2: control 2 ($A\beta_{1-42}$ fibril, $A\beta_{1-42}$ was incubated for 3 h without few-lay Bi_2Se_3), lanes 3-6: $A\beta_{1-42}$ was incubated for 3 h in the presence of few-layer Bi_2Se_3 (12, 60, 300, 1200 ng mL^{-1}, respectively). (d) Relative quantity of $A\beta_{1-42}$ monomer in lane 1-6, which was calculated from c.

Supplementary Table S1 | Effect of few-layer Bi_2Se_3 on secondary structure of $A\beta_{1-42}$. The data of secondary structure of $A\beta_{1-42}$ were calculated from the circular dichroism data using Jasco secondary structure estimation software.

Concentration of few-layer Bi_2Se_3 (ng mL^{-1})	α-helix (%)	β-sheet (%)	β-turn (%)	Random-coil (%)
0	21.0	21.4	25.6	32.1
120	29.9	0.0	30.6	39.5
2400	34.5	0.0	29.0	36.5
$A\beta_{1-42}$ monomer	17.2	0.0	22.0	60.8

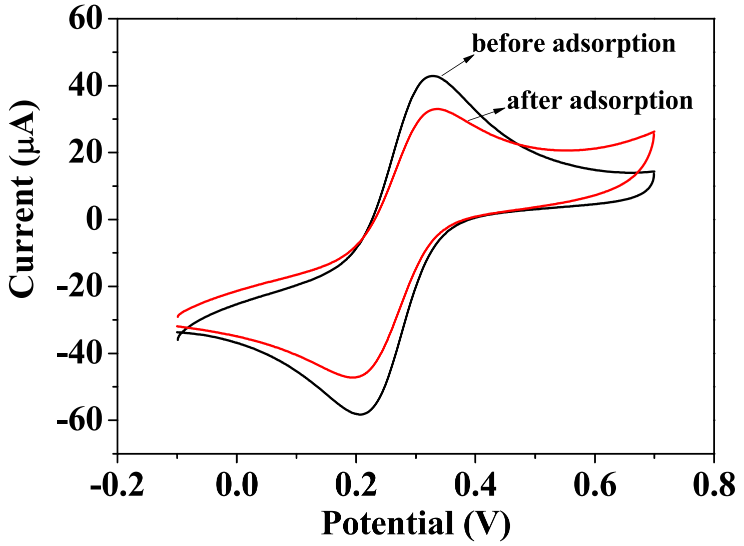

Supplementary Figure S15│CVs of few-layer Bi_2Se_3-modified GCE before and after adsorption of freshly prepared $A\beta_{1\text{-}42}$ monomers.

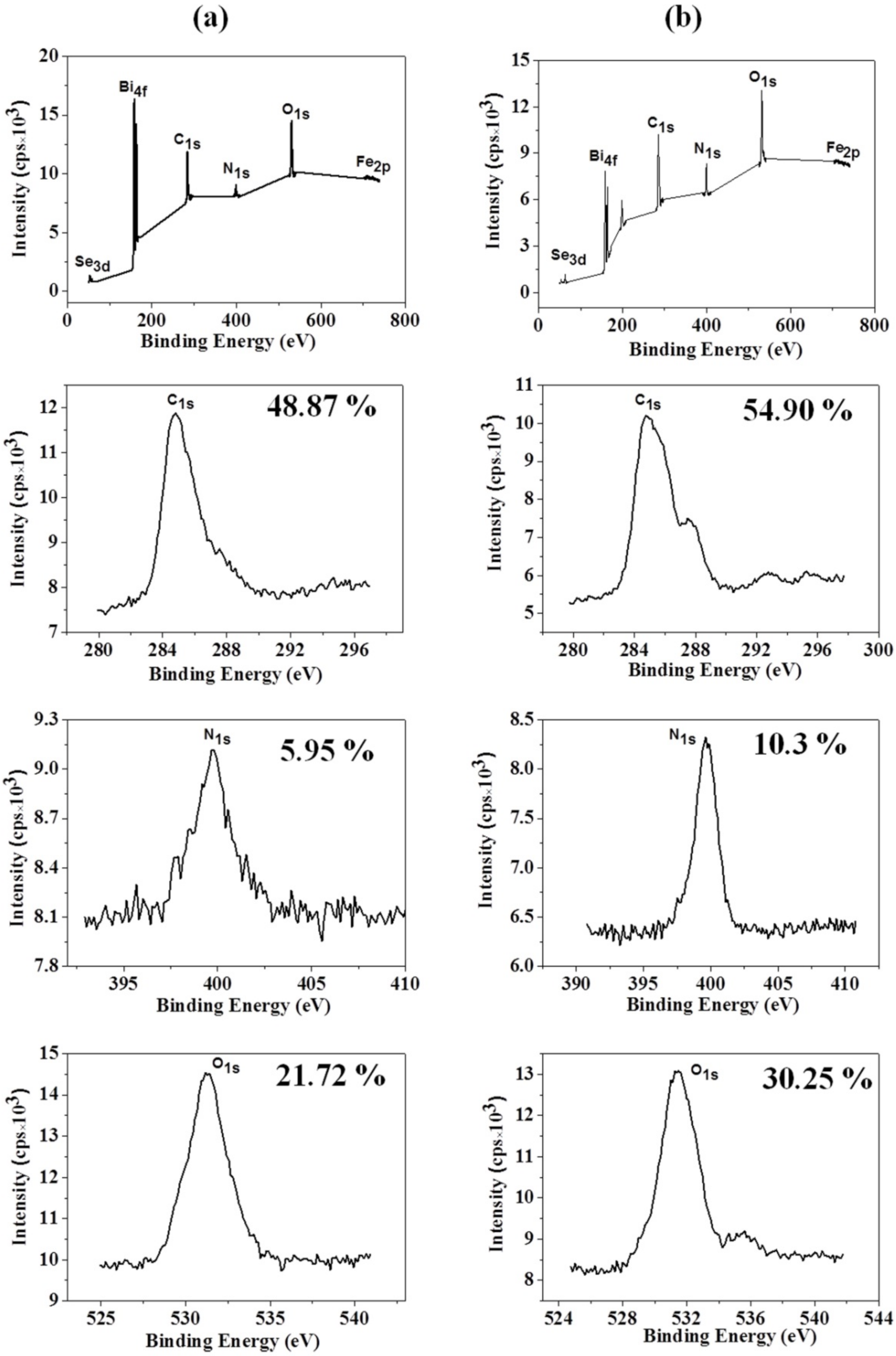

Supplementary Figure S16 | XPS spectra of few-layer Bi_2Se_3 before (a) and after (b) adsorption of $A\beta_{1-42}$ monomers. The contents of carbon, nitrogen and oxygen were indicated in each spectrum.

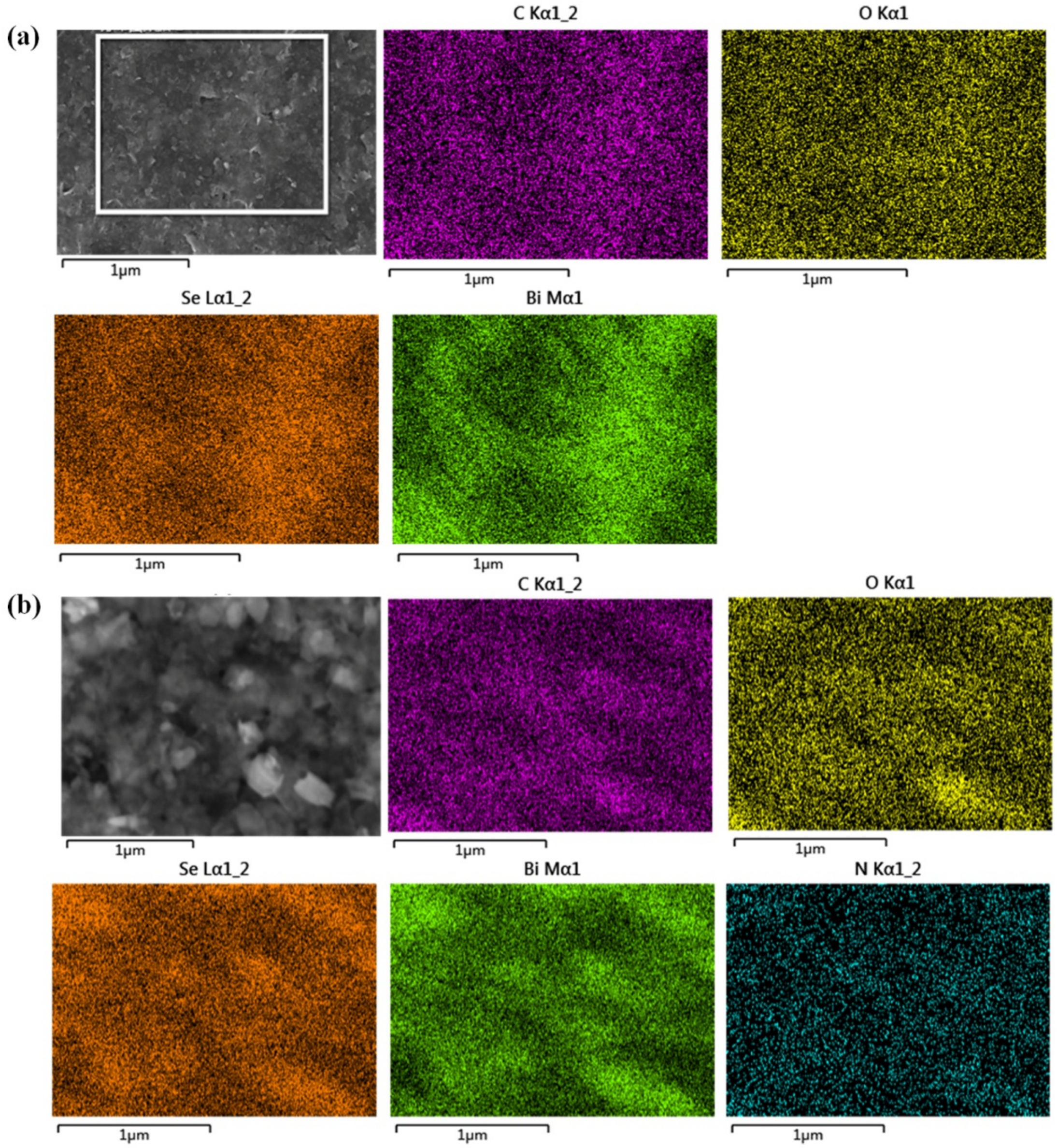

Supplementary Figure S17│SEM images and corresponding quantitative EDS element mapping of few-layer Bi_2Se_3 before (a) and after (b) adsorbing $A\beta_{1-42}$ monomers.

The density and distribution of few-layer Bi_2Se_3 before and after adsorbing $A\beta_{1-42}$ monomers were evaluated by EDS element mapping. Before adsorption (Supplementary Figure S17a), C, O, Bi and Se atoms distribute homogeneously in few-layer Bi_2Se_3 and no N atoms could be observed in the mapping. The N content

might be too low to be detected. However, a new N element appeared and distributed homogeneously on few-layer Bi_2Se_3 after adsorption of $A\beta_{1-42}$ monomers, (Supplementary Figure S17b), indicating that $A\beta_{1-42}$ monomers were adsorbed uniformly onto the surface of few-layer Bi_2Se_3.

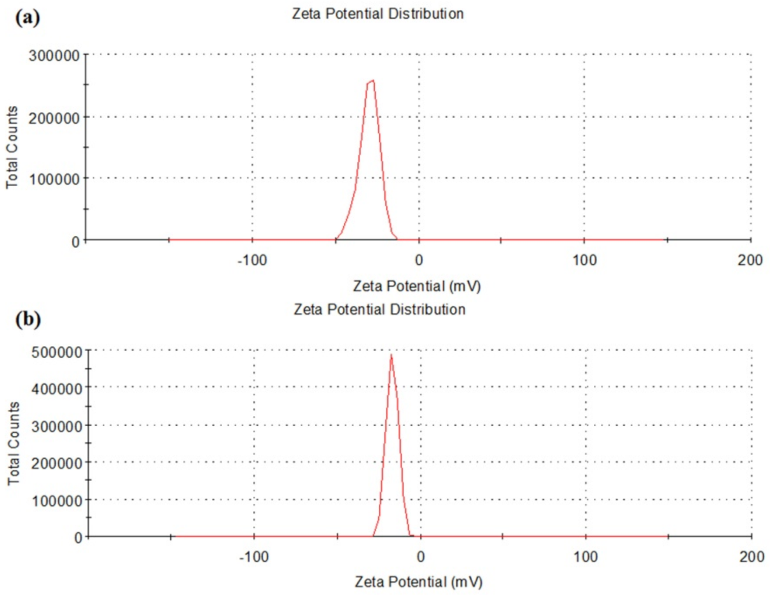

Supplementary Figure S18│Zeta potentials of few-layer Bi_2Se_3 (a) and $A\beta_{1\text{-}42}$ monomer (b) in modified Krebs-Henseliet buffer.

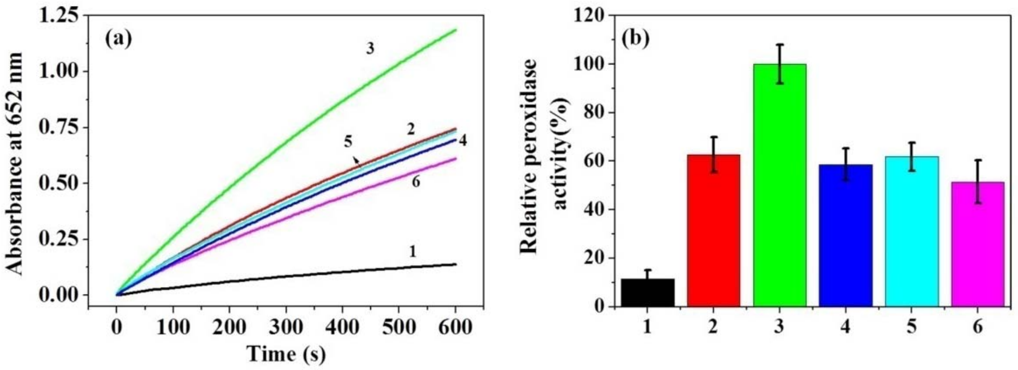

Supplementary Figure S19 | Inhibition of Aβ-mediated peroxidase-like activity by few-layer Bi_2Se_3. Kinetic process (a) and relative peroxidase activity (b) of $Aβ_{1-42}$ (1), hemin (2), hemin + $Aβ_{1-42}$ (3), few-layer Bi_2Se_3 + $Aβ_{1-42}$ incubated at 37 ℃ for 0 h (4) and 3 h (5), and few-layer Bi_2Se_3 (6). The absorption at 652 nm was monitored with time. 3,3',5,5'-Tetramethylbenzidine (TMB) was used as the substrate for peroxidase activity measurement. [Aβ] =50 μM, [hemin] =1.416 μg mL^{-1}, [few-layer Bi_2Se_3] = 12 μg mL^{-1}. [TMB] =0.1 mM, [H_2O_2] =10 mM. Error bars indicate ± s.d. The average of three independent experiments was reported.

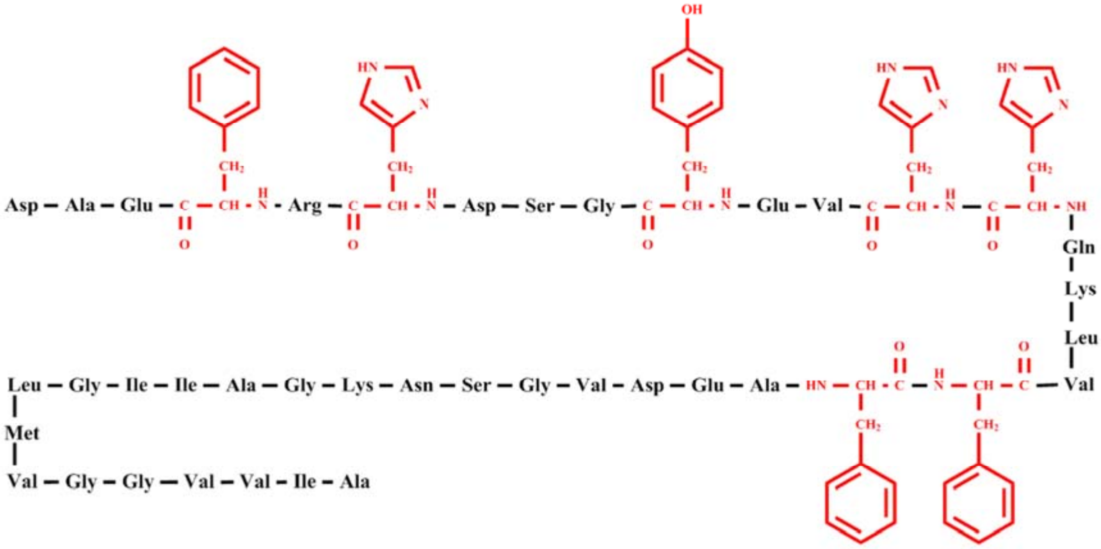

Supplementary Figure S20 | Amino acid sequence of A$\beta_{1\text{–}42}$. Four aromatic amino acids and three heterocyclic amino acids were showed with structural formula in red.
